# Robust SARS-CoV-2-neutralizing antibodies sustained through 6 months post XBB.1.5 mRNA vaccine booster

**DOI:** 10.1016/j.xcrm.2024.101701

**Published:** 2024-08-28

**Authors:** Qian Wang, Ian A. Mellis, Yicheng Guo, Carmen Gherasim, Riccardo Valdez, Aubree Gordon, David D. Ho, Lihong Liu

**Affiliations:** 1 Aaron Diamond AIDS Research Center, Columbia University Vagelos College of Physicians and Surgeons, New York, NY 10032, USA; 2 Pandemic Research Alliance Unit at the Wu Family Center, Columbia University Vagelos College of Physicians and Surgeons, New York, NY 10032, USA; 3 Department of Pathology and Cell Biology, Columbia University Vagelos College of Physicians and Surgeons, New York, NY 10032, USA; 4 Department of Pathology, University of Michigan, Ann Arbor, MI 48109, USA; 5 Department of Epidemiology, University of Michigan, Ann Arbor, MI 48109, USA; 6 Division of Infectious Diseases, Department of Medicine, Columbia University Vagelos College of Physicians and Surgeons, New York, NY 10032, USA; 7 Department of Microbiology and Immunology, Columbia University Vagelos College of Physicians and Surgeons, New York, NY 10032, USA; 8 State Key Laboratory of Virology, College of Life Sciences, Wuhan University, Wuhan, China; 9 Taikang Center for Life and Medical Sciences, Wuhan University, Wuhan, China

**Keywords:** COVID-19, SARS-CoV-2, Omicron subvariant JN.1, XBB.1.5 monovalent mRNA vaccine, neutralizing antibody responses, antibody decay rate

## Abstract

Severe acute respiratory syndrome coronavirus 2 (SARS-CoV-2)-neutralizing antibodies are substantially expanded 1 month after a shot of XBB.1.5 monovalent mRNA vaccine (XBB.1.5 MV) booster, but the durability of this response remains unknown. Here, we address this question by performing neutralization assays on four viral variants (D614G, BA.5, XBB.1.5, and JN.1) using sera from participants obtained at ∼1 month, ∼3 months, and ∼6 months post an XBB.1.5 MV booster. Our findings indicate that the resulting neutralizing antibody titers are robust and generally remain at stable levels for the study period, similar to those following XBB infection. Importantly, this durability of neutralizing antibody titers contrasts with the decline observed after a booster of the original monovalent or BA.5 bivalent mRNA vaccine. Our results are in line with the recent national data from the Centers for Disease Control and Prevention, showing that the efficacy against symptomatic SARS-CoV-2 infection is sustained for up to 4 months after an XBB.1.5 MV booster.

## Highlights

• XBB.1.5 monovalent (MV) booster elicits neutralizing titers up to 6 months after dose
• Titers wane more slowly after XBB.1.5 MV booster than after BA.5 bivalent booster
• XBB.1.5 MV booster partially mitigates immunological imprinting

## Graphical abstract

**Figure:**
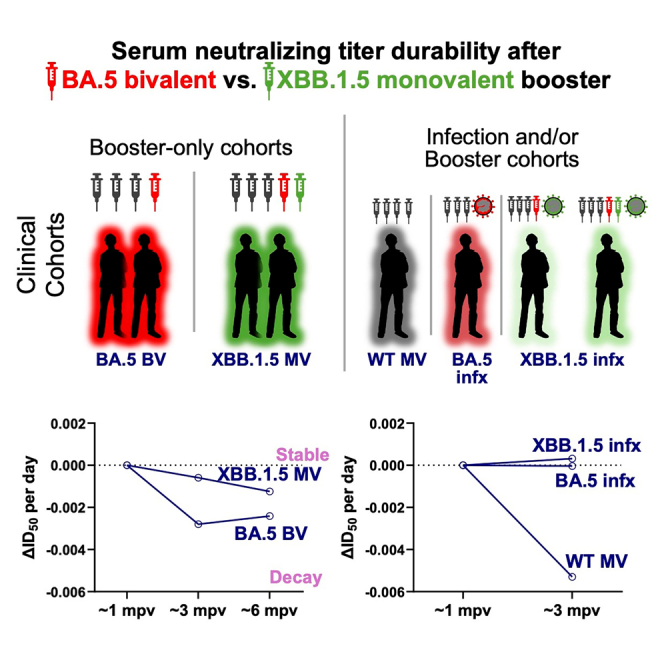

## Teaser

Wang and colleagues find that the XBB.1.5 vaccine booster yields robust serum SARS-CoV-2-neutralizing titers that are stable for ∼6 months, in contrast to results after a booster with previous vaccines but consistent with recent national data showing protection against symptomatic infection lasts for 4 months after the updated vaccine booster.

## Introduction

In the Fall of 2023, the United States Food and Drug Administration authorized new COVID-19 vaccines to replace the previous bivalent vaccines.^1^ The updated vaccines target the spike protein of severe acute respiratory syndrome coronavirus 2 (SARS-CoV-2) Omicron subvariant XBB.1.5, aiming to enhance protection against the viral variant that was most dominant at the time strain selection was made.^2^ Moreover, the updated vaccines were monovalent, because the prior ancestral/BA.5 bivalent vaccines did not appreciably expand the breadth of virus-neutralizing antibodies due to immunological imprinting that biased immune responses toward the ancestral strain.^3^^,^^4^^,^^5^^,^^6^^,^^7^ We and others have since demonstrated that new updated XBB.1.5 mRNA monovalent vaccines (XBB.1.5 MV) boosted the potency and breadth of serum-neutralizing antibodies against not only XBB.1.5 but also other Omicron subvariants, including JN.1, approximately 1 month after administration.^2^^,^^8^^,^^9^^,^^10^^,^^11^^,^^12^^,^^13^^,^^14^^,^^15^ The removal of the ancestral spike from the new vaccine formulations had seemingly mitigated but not eliminated the immunological imprinting observed with the previous bivalent vaccines. However, how the serum antibody responses evolve in the ensuing months after an XBB.1.5 MV booster remains unknown.

## Results

**Figure 1:**
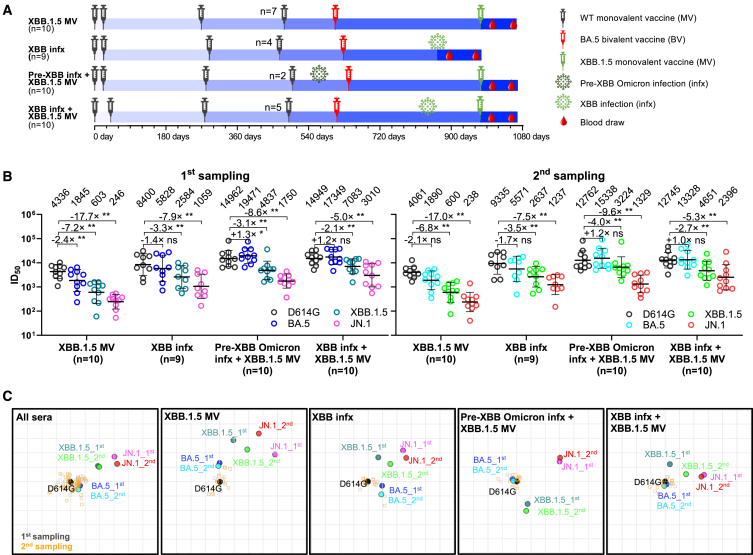
Neutralizing antibody titers in the months after an XBB.1.5 mRNA booster, XBB infection, or both (A) Timelines of vaccine administration, SARS-CoV-2 infection, and serum collection intervals for the four clinical cohorts in this study. Indicated time points represent the mean in days since first SARS-CoV-2 vaccination for each participant; day 0 is defined as the day of the initial SARS-CoV-2 vaccination. All participants previously received 3–4 doses of wild-type (WT) monovalent vaccines (MV), followed by one dose of the BA.5 bivalent vaccine (BV) booster. Numbers of participants for each group receiving a fourth WT MV are indicated. Serum samples were collected from two time points after an XBB.1.5 MV booster or XBB sublineage infection, as indicated, and the mean sample collection days post vaccination or infection are summarized in Table S1. *n*, sample size. (B) Serum virus-neutralizing titers (ID_50_) of the four cohorts against the indicated SARS-CoV-2 pseudoviruses. Geometric mean ID_50_ titers (GMT) are shown along with the fold differences in GMT versus D614G. Statistical analyses comparing GMT between viruses were performed by Wilcoxon matched-pair signed rank tests. ns, not significant; ∗*p* < 0.05; ∗∗*p* < 0.01. (C) Overlaid antigenic maps for serum virus-neutralizing titers at the first (∼26.4 days) and second (∼82.1 days) sampling time points. Panels in order: all cohorts, the XBB.1.5 monovalent vaccine (XBB.1.5 MV) cohort, the XBB infection (XBB infx) cohort, the pre-XBB infection + XBB.1.5 monovalent vaccine (pre-XBB infx + XBB.1.5 MV) cohort, and the XBB infection + XBB.1.5 monovalent vaccine (XBB infx + XBB.1.5 MV) cohort. Each panel contains two overlaid antigenic maps generated using sera from two time points post XBB exposure independently, with the D614G variant aligned. The x-y orientation of the component maps for either first sampling or second sampling is free, as only the relative distances between the variants within a sampling and the respective sampling’s sera are compared. Distances between first sampling and second sampling variant points are not directly compared. One grid square on the antigenic maps corresponds to one antigenic unit, representing an approximately 2-fold change in ID_50_ titer. Variant positions are indicated by circles, while serum positions are denoted by gray squares (first sampling) or orange squares (second sampling). See also Tables S1 and S2.

First, we addressed this question by evaluating the serum virus-neutralizing titers at two distinct time points in 39 participants distributed across four cohorts: (1) who had received an XBB.1.5 MV booster without history of SARS-CoV-2 infection (XBB.1.5 MV); (2) who had XBB sublineage virus infection without history of XBB.1.5 MV booster (XBB infection [XBB infx]); (3) who had received an XBB.1.5 MV booster with a prior pre-XBB Omicron infection history (pre-XBB Omicron infx + XBB.1.5 MV); and (4) who had received an XBB.1.5 MV booster with a prior XBB sublineage infection history (XBB infx + XBB.1.5 MV) (Figure 1A). The first and second serum samplings occurred at 26.4 and 82.1 mean days post vaccination or infection, respectively (Figure 1A; Table S1). Demographics and vaccination histories among the four clinical cohorts are presented in Tables S1 and S2. Serum-neutralizing antibody titers were determined using vesicular stomatitis virus-based pseudoviruses expressing the spike proteins of D614G (ancestral), BA.5, XBB.1.5, or JN.1.

Overall, similar patterns in neutralizing antibody titers (ID_50_) were observed between serum samples from the first and second time points for all four cohorts (Figure 1B), with several features worthy of emphasis. First, neutralizing titers against all four viruses tested were robust (≥256) at both time points. Second, the highest neutralizing titers of the XBB.1.5 MV cohort were observed against D614G, while titers against XBB.1.5 were much lower (4,647 versus 646 and 4,535 versus 643 at first and second time points, respectively). This finding showed the persistence of immunological back-boosting (also known as immunological imprinting) in eliciting neutralizing antibodies against SARS-CoV-2, although not as severe as previously observed for the ancestral/BA.5 bivalent vaccine booster.^3^^,^^4^^,^^5^^,^^6^^,^^7^ Third, as expected, sera of participants from “pre-XBB Omicron infx + XBB.1.5 MV” and “XBB infx + XBB.1.5 MV” cohorts (individuals who had one more exposure to Omicron spike) showed substantially higher neutralizing titers against all four viruses. Fourth, sera from the XBB infx cohort exhibited stronger neutralizing activity (1.8- to 4.8-fold) than sera from the XBB.1.5 MV cohort. Lastly, among the viruses tested, serum-neutralizing titers were the lowest for JN.1 in all cohorts, indicating that it is the most antibody evasive, in line with our previous report.^2^

Antigenic maps were then generated using serum neutralization data from the four cohorts, both collectively and individually (Figure 1C), to visually summarize our findings. These maps showed that the antigenic distances between the ancestral D614G virus and other tested SARS-CoV-2 Omicron subvariants at both time points were consistent across all cohorts. Infections with an XBB sublineage virus seemed to slightly outperform a single XBB.1.5 MV booster in reducing antigenic distance. Additionally, having a history of Omicron infection prior to XBB.1.5 MV booster enhanced the neutralization of the currently dominant JN.1 subvariant. In particular, exposure to XBB sublineage infection before the booster appeared to reduce the antigenic distance to JN.1 by approximately one antigenic unit more than a pre-XBB Omicron infection.

**Figure 2:**
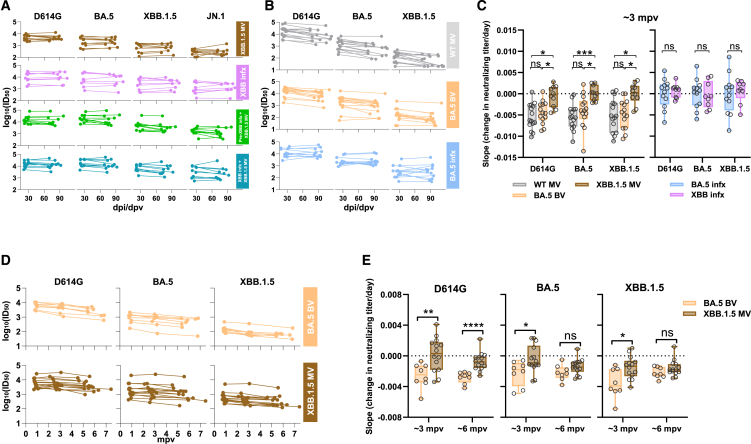
An XBB.1.5 monovalent vaccine booster induced sustained virus-neutralizing antibody responses through 6 months post vaccination (A) Comparison of serum neutralizing antibody titers between two time points after XBB vaccination or infection for each participant in each cohort. (B) Comparison of serum-neutralizing antibody titers between two time points after the fourth dose of the WT MV or BA.5 BV, or BA.5 breakthrough infection. Data for “WT MV,” “BA.5 BV,” and “BA.5 infx” cohorts were extracted from a previously published study.^4^ (C) Estimated change in neutralizing titer (log_10_(ID_50_)) per day (i.e., the slopes for serum samples collected from approximately 1 to 3 months) for cohorts without SARS-CoV-2 infection (left) or with breakthrough infection (right) across the two respective studies. Negative values indicate the rate of titer decay. (D) Decline in neutralizing antibody titers over approximately a 6-month period in individuals who received either the XBB.1.5 monovalent vaccine booster or the BA.5 bivalent vaccine booster. mpv, month post vaccination. (E) Estimated change in neutralizing titer (log_10_(ID_50_)) per day for longitudinal cohorts is presented in (D). Negative values indicate the rate of titer decay. Box-and-whisker plots, with whisker limits at minimum and maximum values and the central line representing median. Statistical analyses were performed by Mann-Whitney U tests. ns, not significant; ∗*p* < 0.05; ∗∗*p* < 0.01; ∗∗∗*p* < 0.001; ∗∗∗∗*p* < 0.0001. See also Figure S1 and Table S3.

We next endeavored to better understand the change in virus-neutralizing titers between the first and second time point serum samples. The ID_50_ titers against four viruses for all four cohorts were plotted using the actual dates of serum collection post vaccination or infection (Figure 2A). The daily decline in neutralizing titers (median slopes) is summarized in Figure S1A. No significant waning of neutralizing titers was observed for all four cohorts, except for a slight decrease in titer against XBB.1.5 in the pre-XBB infx + XBB.1.5 MV cohort (1.5-fold decrease). Additionally, no differences in antibody titer declines were found among all four cohorts against each tested virus. However, in our prior study,^4^ we observed around a 2-fold or greater decrease in neutralizing titers against D614G, BA.5, and XBB.1.5 from ∼1 month to ∼3 months post the fourth dose of the wild-type monovalent vaccine (WT MV) booster or ancestral/BA.5 bivalent vaccine (BV) booster, while a BA.5 breakthrough infection led to stable serum-neutralizing activity over the same interval (Figure 2B). Since the sample collection times across the two studies were slightly different (Table S1; Figure S1B), the most appropriate comparison of antibody decline would be to determine the slope between the log_10_(ID_50_) titers for the two time points. Comparing cohorts with vaccine boosters but without any history or laboratory evidence of prior SARS-CoV-2 infection, the median slope for all four viruses tested was nearly zero (flat) after an XBB.1.5 MV booster in the present study, whereas the median slopes were significantly more negative (decline, with median values ranging from −0.0040/day to −0.0063/day) after a WT MV or ancestral/BA.5 BV booster in a prior study (Figure 2C). On the other hand, there was no appreciable decline during the study period for serum-neutralizing titers for individuals who had a BA.5 or XBB breakthrough infection in both studies (Figure 2C).

Lastly, in order to track antibody dynamics through time, we conducted a follow-up study, extending to approximately 6 months, for the exposure histories of two specific groups: 14 individuals vaccinated with an XBB.1.5 monovalent vaccine booster and 8 individuals with a BA.5 bivalent vaccine booster. Due to the limited availability of samples, most XBB.1.5 MV samples were obtained from additional individuals enrolled in a related study. Clinical details for all participants in the 6-month analysis are listed in Table S3. Consistent with the observation from the 3-month samples, the waning of titers until the 6-month time point was slower for those receiving the XBB.1.5 monovalent vaccine than for those receiving the BA.5 bivalent vaccine (Figures 2D and 2E).

## Discussion

In summary, an XBB.1.5 MV booster has elicited robust and sustained neutralizing antibodies against SARS-CoV-2, including the currently dominant JN.1 subvariant, for several months (Figures 1 and 2). The reduced waning of neutralizing antibodies is in distinct contrast to that observed after an ancestral MV or ancestral/BA.5 BV booster (Figure 2C). A clear explanation for this discrepancy is lacking. There is no indication that we may have missed the peak antibody response with the first serum sampling (Figure S1B). While an SARS-CoV-2 infection between the two time points could not be excluded, antibodies directed to the viral nucleocapsid could not be detected in the sera from the XBB.1.5 MV cohort (data not shown), nor is there a clinical history consistent with an intervening infection. The short-term durability of the potency and breadth of the virus-neutralization response following XBB.1.5 MV booster again suggests that immunological imprinting has been partially mitigated, likely due to the exclusion of the ancestral spike from the updated vaccine formulation. However, it should be noted that immunological imprinting has not been completely overridden by two exposures to an Omicron spike, as evident by higher neutralizing antibody titers against D614G compared to XBB.1.5 in the two cohorts that had an Omicron infection followed by an XBB.1.5 MV booster (Figure 1B). Importantly, our data are in agreement with vaccine effectiveness estimates recently reported by the Center for Disease Control and Prevention, showing no appreciable decline in protection against symptomatic COVID-19 up to 4 months post booster with updated vaccines.^16^

### Limitations of the study

There are several notable limitations to this study. First, the number of study subjects in each cohort is relatively small, even though statistically significant results are reported. Second, we still lack an explanation for the mechanism by which neutralizing antibody titers after an XBB.1.5 MV booster are more stable than those elicited by earlier vaccinations. More studies are required to provide an answer. Third, the antibody responses assessed here, including back-boosting of titers against earlier SARS-CoV-2 variants, are exclusively serum-neutralizing antibodies. It would be beneficial for future research to encompass a variety of studies, such as antibody binding, memory B cell assays, and T cell analysis, to shed light on the specific mechanisms underlying immunological imprinting.

## STAR★Methods

### Key resources table

**Table:** 

REAGENT or RESOURCE	SOURCE	IDENTIFIER
**Bacterial and virus strains**		

VSV-G pseudotyped ΔG-luciferase	Kerafast	Cat# EH1020-PM

**Biological samples**		

“XBB.1.5 MV” sera	This paper	N/A
“XBB infx” sera	This paper	N/A
“Pre-XBB infx + XBB.1.5 MV” sera	This paper	N/A
“XBB infx + XBB.1.5 MV” sera	This paper	N/A
“WT MV” sera	Wang et al.^4^	N/A
“BA.5 MV” sera	This paper and Wang et al.^4^	N/A

**Chemicals, peptides, and recombinant proteins**		

Polyethylenimine (PEI)	Polysciences Inc.	Cat# 23966-100

**Critical commercial assays**		

Luciferase Assay System	Promega	Cat# E4550

**Experimental models: Cell lines**		

HEK293T	ATCC	Cat# CRL-3216; RRID: CVCL_0063
Vero-E6	ATCC	Cat# CRL-1586; RRID: CVCL_0574

**Recombinant DNA**		

pCMV3-D614G	Wang et al.^17^	N/A
pCMV3-BA.5	Wang et al.^17^	N/A
pCMV3-XBB.1.5	Wang et al.^5^	N/A
pCMV3-JN.1	Wang et al.^2^	N/A

**Software and algorithms**		

GraphPad Prism V.10	GraphPad Software Inc	https://www.graphpad.com/scientific-software/prism/

### Resource availability

#### Lead contact

Further information and requests for resources and reagents should be directed to and will be fulfilled by the lead contact, Lihong Liu (llh3411@whu.edu.cn).

#### Materials availability

All reagents generated in this study are available from the lead contact author with a completed Materials Transfer Agreement.

#### Data and code availability

Data:

Data reported in this paper will be shared by the lead contact author upon request.

Code:

This paper does not report original code. Any additional information required to reanalyze the data reported in this paper and R scripts (version 1.1.4, available at https://acorg.github.io/Racmacs/) used to perform most statistical analyses are available from the lead contact author upon request.

### Experimental model and subject details

#### Clinical cohorts

Longitudinal sera were obtained as part of an ongoing cohort study, Immunity-Associated with SARS-CoV-2 Study (IASO), which began in 2020 at the University of Michigan in Ann Arbor, Michigan.^18^ All participants provided written informed consent, and sera were collected according to the protocol approved by the Institutional Review Board of the University of Michigan Medical School. Participants in the IASO study completed weekly symptom surveys, and if any symptoms were reported, participants were tested for SARS-CoV-2. We tested all serum samples by anti-nucleoprotein (NP) ELISA to check for any potential breakthrough infections during the period spanning sample collections for this study.

In this study, we included sera from 39 individuals across four clinical cohorts: 1) individuals with no recorded SARS-CoV-2 infections who had received an XBB.1.5 monovalent vaccine booster (“XBB.1.5 MV”); 2) individuals with a recent XBB sublineage infection who had not received the XBB.1.5 booster (“XBB infx); 3) individuals with pre-XBB Omicron infection who also received the XBB.1.5 booster (“Pre-XBB Omicron infx + XBB MV”); and 4) individuals with an XBB sublineage infection who also received the XBB.1.5 booster (XBB infx + XBB MV). Individuals in all cohorts previously received either three or four doses of a wildtype monovalent vaccine as well as a single ancestral/BA.5 bivalent booster. Most participants were female (79.5%) with an average age of 51.6 years. Sera were collected an average of 26.4 and 82.1 days after XBB.1.5 vaccination or XBB sublineage infection. Demographic, vaccination, and serum collection details are summarized for each cohort in Table S1, and details are shown for each participant in Table S2. Additionally, we collected longitudinal serum samples at approximately 1-month, 3-month, and 6-month intervals from 14 individuals vaccinated with an XBB.1.5 monovalent vaccine booster and 8 individuals with a BA.5 bivalent vaccine booster. Clinical details for all cases are provided in Table S3.

#### Cell lines

We obtained 293T (CRL-3216) and Vero-E6 (CRL-1586) cells from ATCC and cultured them in a humidified incubator at 37°C, supplemented with 5% CO_2_. The morphology of each cell line was visually confirmed before use. All cell lines tested negative for mycoplasma. The research resource identifiers (RRIDs) for the cell lines used in our study are listed in the key resources table.

### Method details

#### Pseudovirus neutralization assay

Plasmids encoding SARS-CoV-2 variant spikes, including D614G, BA.5, XBB.1.5, and JN.1, were generated in previous studies.^2^^,^^5^^,^^17^

To produce pseudotyped viruses of SARS-CoV-2 variants, we transfected 293T cells with the spike-encoding plasmids described above using 1 mg/mL PEI (Polyethylenimine). One day post-transfection, the 293T cells were incubated with VSVG∗ΔG-luciferase (Kerafast, Inc.) at a multiplicity of infection of approximately 3–5 for 2 h followed by three washes with complete culture medium. The cells were then cultured with fresh medium for an additional day. Cell supernatants containing viruses were collected, clarified by centrifugation, aliquoted, and stored at −80°C until use.

The viral titer of each variant was titrated to calculate a 50% tissue-culture-infectious dose (TCID_50_) and normalized for neutralization assays. Serum samples were diluted in triplicate in 96-well plates, starting from a 12.5-fold dilution (for one sample, a 50-fold starting dilution was necessary due to volume constraints), and then incubated with an equal volume of virus for 1 h at 37°C before adding 2 × 10^4^ cells/well of Vero-E6 cells. The cells were then cultured overnight, harvested, and lysed for measurement of luciferase activity using SoftMax Pro v.7.0.2 (Molecular Devices). Reductions in luciferase activity at given dilutions of sera were calculated, and ID_50_ values of sera were obtained by fitting the virus-reduction data using a non-linear five-parameter dose-response curve in GraphPad Prism V.10.

#### Antigenic cartography

Antigenic maps for D614G and other SARS-CoV-2 variants were generated by integrating the ID_50_ values of individual serum samples using a published antigenic cartography method.^19^ Visualizations were created with the Racmacs package (version 1.1.4, available at https://acorg.github.io/Racmacs/) in R software version 4.0.3. The optimization was performed over 2,000 steps, with the “minimum column basis” parameter set to “none”. The “mapDistances” function calculated the antigenic distances between each serum sample and each variant. D614G was used as center for each group’s sera, and the seeds for each antigenic map were manually adjusted to ensure that XBB.1.5 and JN.1 were oriented correctly relative to D614G. All maps are aligned with D614G positioned in the middle left, ensuring a consistent reference point across the maps.

### Quantification and statistical analysis

Serum neutralization ID_50_ values were calculated using a five-parameter dose-response curve in GraphPad Prism v.10. Evaluations of statistical significance were performed employing either Wilcoxon matched-pairs signed rank tests or Mann-Whitney U tests using GraphPad Prism v.10 software.

## Supplemental information

Document S1. Figure S1 and Tables S1–S3

Document S2. Article plus supplemental information
