# Supplementary material for: Robust SARS-CoV-2-neutralizing antibodies sustained through 6 months post XBB.1.5 mRNA vaccine booster
Source: Cell Rep Med. 2024 Aug 28;5(9):101701. doi: 10.1016/j.xcrm.2024.101701 (PMC11524932; doi:10.1016/j.xcrm.2024.101701)
Supplement: Document S1. Figure S1 and Tables S1–S3 [file mmc1.pdf]

**Cell Reports Medicine, Volume *5***

## Supplemental information

# Robust SARS-CoV-2-neutralizing antibodies sustained through 6 months post XBB.1.5 mRNA vaccine booster

**Qian Wang, Ian A. Mellis, Yicheng Guo, Carmen Gherasim, Riccardo Valdez, Aubree Gordon, David D. Ho, and Lihong Liu**

| Clinical Information | | Across all | | XBB.1.5 MV | | XBB infx | | Prior Omicron infection + XBB.1.5 MV | | | |
|---|---|---|---|---|---|---|---|---|---|---|---|
| | | | | | | | | Pre-XBB infx + XBB.1.5 MV | | XBB infx + XBB.1.5 MV | |
| | | Number or mean | % or range | Number or mean | % or range | Number or mean | % or range | Number or mean | % or range | Number or mean | % or range |
| **Total participants** | | 39 | | 10 | | 9 | | 10 | | 10 | |
| **Male** | | 8 | 20.5% | 3 | 30% | 1 | 11.10% | 2 | 20% | 2 | 20% |
| **Female** | | 31 | 79.5% | 7 | 70% | 8 | 88.90% | 8 | 80% | 8 | 80% |
| **Age** | | 51.6 | (30, 67) | 52.6 | (38, 65) | 52.1 | (40, 59) | 49.7 | (36, 67) | 52.1 | (30, 62) |
| **First two WT doses** | **Pfizer** | 35 | 89.7% | 9 | 90% | 8 | 88.90% | 9 | 90% | 9 | 90% |
| | **Moderna** | 3 | 7.7% | 1 | 10% | 1 | 11.10% | 1 | 10% | 0 | 0% |
| | **Janssen** | 1 | 2.6% | 0 | 0% | 0 | 0% | 0 | 0% | 1 | 10% |
| **Third WT dose** | **Pfizer** | 34 | 87.2% | 9 | 90% | 8 | 88.90% | 9 | 90% | 8 | 80% |
| | **Moderna** | 5 | 12.8% | 1 | 10% | 1 | 11.10% | 1 | 10% | 2 | 20% |
| **Fourth WT dose** | **Pfizer** | 13 | 33.3% | 4 | 40% | 3 | 33.30% | 2 | 20% | 4 | 40% |
| | **Moderna** | 5 | 12.8% | 3 | 30% | 1 | 11.10% | 0 | 0% | 1 | 10% |
| | **None** | 21 | 53.8% | 3 | 30% | 5 | 55.60% | 8 | 80% | 5 | 50% |
| **BA.5 bivalent booster** | **Pfizer** | 23 | 59% | 6 | 60% | 7 | 77.80% | 4 | 40% | 6 | 60% |
| | **Moderna** | 16 | 41% | 4 | 40% | 2 | 22.20% | 6 | 60% | 4 | 40% |
| **XBB MV booster** | **Pfizer** | 13 | 33.3% | 4 | 40% | 0 | 0% | 3 | 30% | 6 | 60% |
| | **Moderna** | 17 | 43.6% | 6 | 60% | 0 | 0% | 7 | 70% | 4 | 40% |
| | **None** | 9 | 23.1% | 0 | 0% | 9 | 100% | 0 | 0% | 0 | 0% |
| **First sample post-XBB (days)** | | 26.4 | (21, 34) | 26.2 | (22, 32) | 27.8 | (24, 30) | 26.9 | (21, 34) | 24.7 | (21, 30) |
| **Second sample post-XBB (days)** | | 82.1 | (68, 104) | 82.1 | (70, 93) | 92.1 | (78, 104) | 77.6 | (68, 97) | 77.5 | (68, 92) |

**Table S1. Summary of information on participants in this study who were exposed to XBB. Related to Figure 1.** Demographic, vaccine, and serum collection information summarized for each cohort. Listed values represent the mean and range (age and sera collection) or number and percentage (vaccine type and sex).

| Participant ID | Age | Gender | Race | Ethnicity | Infection Period | WT vaccines | | | | Bivalent vaccine | XBB.1.5 MV vaccine | Days post-XBB | | Days after 1st WT vaccine dose | | | | |
|---|---|---|---|---|---|---|---|---|---|---|---|---|---|---|---|---|---|---|
| | | | | | | 1st dose | 2nd dose | 3rd dose | 4th dose | | | 1st sample | 2nd sample | 2nd WT dose | 3rd WT dose | 4th WT dose | Bivalent vaccine | XBB.1.5 MV vaccine |
| **XBB.1.5 MV (n=12)** | | | | | | | | | | | | | | | | | | |
| G4-1 | 62 | Female | White | Not Hispanic or Latino | NA | Pfizer | Pfizer | Pfizer | Pfizer | Pfizer | Moderna | 27 | 93 | 22 | 268 | 491 | 657 | 993 |
| G4-4 | 65 | Female | White | Not Hispanic or Latino | NA | Pfizer | Pfizer | Pfizer | Pfizer | Pfizer | Pfizer | 28 | 79 | 22 | 266 | 503 | 637 | 990 |
| G4-6 | 55 | Female | White | Not Hispanic or Latino | NA | Pfizer | Pfizer | Pfizer | Moderna | Moderna | Moderna | 25 | 82 | 21 | 272 | 472 | 637 | 995 |
| G4-8 | 38 | Male | White | Not Hispanic or Latino | NA | Moderna | Moderna | Moderna | NA | Moderna | Pfizer | 27 | 88 | 28 | 235 | NA | 539 | 914 |
| G4-9 | 64 | Female | White | Not Hispanic or Latino | NA | Pfizer | Pfizer | Pfizer | Pfizer | Pfizer | Pfizer | 32 | 70 | 21 | 270 | 455 | 606 | 976 |
| G4-12 | 40 | Male | White | Not Hispanic or Latino | NA | Pfizer | Pfizer | Pfizer | NA | Moderna | Moderna | 22 | 78 | 21 | 268 | NA | 584 | 969 |
| G4-14 | 50 | Male | White | Not Hispanic or Latino | NA | Pfizer | Pfizer | Pfizer | Moderna | Pfizer | Moderna | 31 | 88 | 21 | 235 | 444 | 621 | 996 |
| G4-15 | 54 | Female | White | Not Hispanic or Latino | NA | Pfizer | Pfizer | Pfizer | Moderna | Pfizer | Moderna | 22 | 89 | 21 | 329 | 490 | 623 | 990 |
| G4-17 | 56 | Female | White | Not Hispanic or Latino | NA | Pfizer | Pfizer | Pfizer | Pfizer | Pfizer | Moderna | 22 | 71 | 21 | 287 | 507 | 654 | 1016 |
| G4-19 | 42 | Female | White | Not Hispanic or Latino | NA | Pfizer | Pfizer | Pfizer | NA | Moderna | Pfizer | 26 | 83 | 21 | 239 | NA | 520 | 910 |
| **XBB infx (n=9)** | | | | | | | | | | | | | | | | | | |
| G3-4 | 59 | Female | White | Not Hispanic or Latino | 2023.08 | Pfizer | Pfizer | Pfizer | Pfizer | Pfizer | NA | 26 | 78 | 21 | 295 | 472 | 660 | NA |
| G3-6 | 54 | Female | White | Not Hispanic or Latino | 2023.09 | Pfizer | Pfizer | Pfizer | Pfizer | Pfizer | NA | 28 | 100 | 21 | 222 | 421 | 602 | NA |
| G3-9 | 59 | Female | White | Not Hispanic or Latino | 2023.05 | Pfizer | Pfizer | Pfizer | Pfizer | Pfizer | NA | 30 | 86 | 25 | 281 | 469 | 613 | NA |
| G3-12 | 42 | Male | White | Not Hispanic or Latino | 2023.04 | Pfizer | Pfizer | Pfizer | NA | Moderna | NA | 28 | 88 | 21 | 269 | NA | 615 | NA |
| G3-15 | 53 | Female | White | Not Hispanic or Latino | 2023.02 | Pfizer | Pfizer | Pfizer | NA | Pfizer | NA | 27 | 97 | 22 | 320 | NA | 645 | NA |
| G3-16 | 40 | Female | White | Not Hispanic or Latino | 2023.05 | Pfizer | Pfizer | Pfizer | NA | Pfizer | NA | 28 | 90 | 21 | 279 | NA | 658 | NA |
| G3-17 | 49 | Female | White | Not Hispanic or Latino | 2023.07 | Pfizer | Pfizer | Pfizer | NA | Moderna | NA | 30 | 95 | 22 | 328 | NA | 641 | NA |
| G3-18 | 59 | Female | White | Not Hispanic or Latino | 2023.04 | Moderna | Moderna | Moderna | Moderna | Pfizer | NA | 24 | 91 | 28 | 268 | 505 | 584 | NA |
| G3-20 | 54 | Female | White | Not Hispanic or Latino | 2023.08 | Pfizer | Pfizer | Pfizer | NA | Pfizer | NA | 29 | 104 | 23 | 284 | NA | 632 | NA |
| **Pre-XBB infx + XBB.1.5 MV (n=10)** | | | | | | | | | | | | | | | | | | |
| G5-1 | 41 | Female | Declined | Not Hispanic or Latino | 2022.09 | Moderna | Moderna | Moderna | NA | Pfizer | Moderna | 25 | 68 | 28 | 330 | NA | 715 | 1009 |
| G5-2 | 61 | Female | White | Not Hispanic or Latino | 2022.04 | Pfizer | Pfizer | Pfizer | NA | Moderna | Moderna | 34 | 76 | 22 | 302 | NA | 649 | 1006 |
| G5-3 | 53 | Female | White | Not Hispanic or Latino | 2022.04 | Pfizer | Pfizer | Pfizer | NA | Pfizer | Moderna | 25 | 81 | 21 | 275 | NA | 626 | 999 |
| G5-4 | 49 | Male | White | Not Hispanic or Latino | 2022.01 | Pfizer | Pfizer | Pfizer | NA | Pfizer | Pfizer | 22 | 77 | 21 | 265 | NA | 691 | 956 |
| G5-5 | 67 | Female | White | Not Hispanic or Latino | 2022.07 | Pfizer | Pfizer | Pfizer | Pfizer | Moderna | Moderna | 29 | 73 | 21 | 271 | 473 | 690 | 992 |
| G5-7 | 48 | Female | White | Not Hispanic or Latino | 2022.01 | Pfizer | Pfizer | Pfizer | NA | Moderna | Pfizer | 22 | 75 | 21 | 254 | NA | 554 | 925 |
| G5-10 | 40 | Female | Multiple | Not Hispanic or Latino | 2022.04 | Pfizer | Pfizer | Pfizer | NA | Moderna | Moderna | 21 | 70 | 24 | 276 | NA | 638 | 1003 |
| G5-12 | 43 | Female | White | Not Hispanic or Latino | 2022.01 | Pfizer | Pfizer | Pfizer | NA | Moderna | Pfizer | 32 | 72 | 21 | 234 | NA | 566 | 913 |
| G5-13 | 36 | Male | White | Not Hispanic or Latino | 2022.08 | Pfizer | Pfizer | Pfizer | NA | Moderna | Moderna | 30 | 97 | 21 | 316 | NA | 655 | 980 |
| G5-14 | 59 | Female | White | Not Hispanic or Latino | 2022.04 | Pfizer | Pfizer | Pfizer | Pfizer | Pfizer | Moderna | 29 | 87 | 21 | 277 | 543 | 610 | 986 |
| **XBB infx + XBB.1.5 MV (n=10)** | | | | | | | | | | | | | | | | | | |
| G6-1 | 54 | Female | White | Not Hispanic or Latino | 2023.02 | Pfizer | Pfizer | Pfizer | Moderna | Pfizer | Moderna | 22 | 70 | 21 | 262 | 492 | 632 | 1003 |
| G6-2 | 58 | Female | White | Not Hispanic or Latino | 2023.02 | Pfizer | Pfizer | Pfizer | NA | Pfizer | Pfizer | 22 | 74 | 21 | 284 | NA | 682 | 1000 |
| G6-3 | 61 | Male | White | Not Hispanic or Latino | 2023.05 | Pfizer | Pfizer | Moderna | NA | Moderna | Moderna | 30 | 87 | 21 | 231 | NA | 541 | 917 |
| G6-4 | 42 | Female | White | Not Hispanic or Latino | 2023.03 | Pfizer | Pfizer | Pfizer | Pfizer | Moderna | Pfizer | 29 | 83 | 21 | 266 | 477 | 608 | 996 |
| G6-5 | 62 | Female | White | Not Hispanic or Latino | 2023.06 | Pfizer | Pfizer | Pfizer | Pfizer | Pfizer | Pfizer | 21 | 73 | 21 | 277 | 439 | 643 | 1002 |
| G6-6 | 46 | Male | White | Not Hispanic or Latino | 2023.05 | Janssen | Janssen | Moderna | NA | Pfizer | Pfizer | 24 | 71 | 214 | 386 | NA | 543 | 921 |
| G6-7 | 61 | Female | White | Not Hispanic or Latino | 2023.04 | Pfizer | Pfizer | Pfizer | Pfizer | Moderna | Moderna | 26 | 76 | 21 | 275 | 519 | 625 | 1004 |
| G6-8 | 62 | Female | Asian | Not Hispanic or Latino | 2023.04 | Pfizer | Pfizer | Pfizer | Pfizer | Pfizer | Moderna | 29 | 81 | 21 | 294 | 516 | 630 | 1000 |
| G6-9 | 30 | Female | White | Hispanic or Latino | 2023.08 | Pfizer | Pfizer | Pfizer | NA | Moderna | Pfizer | 22 | 68 | 21 | 218 | NA | 580 | 922 |
| G6-11 | 45 | Female | White | Not Hispanic or Latino | 2023.08 | Pfizer | Pfizer | Pfizer | NA | Pfizer | Pfizer | 22 | 92 | 21 | 269 | NA | 624 | 984 |

**Table S2. Details of participants exposed to XBB. Related to Figure 1.** Clinical information for each participant, including demographic, vaccine, infection, and sera details.

| Participant ID | Age | Gender | Race | Ethnicity | Infection Period | Days post-vaccination | | |
|---|---|---|---|---|---|---|---|---|
| | | | | | | 1st sample | 2nd sample | 3rd sample |
| **BA.5 BV (n=8)** | | | | | | | | |
| BA.5-1 | 24 | Female | White | Not Hispanic or Latino | NA | 30 | 85 | 162 |
| BA.5-2 | 43 | Female | White | Not Hispanic or Latino | NA | 26 | 91 | 205 |
| BA.5-3 | 45 | Male | White | Not Hispanic or Latino | NA | 26 | 98 | 204 |
| BA.5-4 | 36 | Female | White | Not Hispanic or Latino | NA | 27 | 90 | 173 |
| BA.5-5 | 38 | Female | White | Not Hispanic or Latino | NA | 24 | 90 | 198 |
| BA.5-6 | 36 | Male | White | Not Hispanic or Latino | NA | 24 | 95 | 161 |
| BA.5-7 | 42 | Female | White | Not Hispanic or Latino | NA | 27 | 97 | 177 |
| BA.5-8 | 32 | Female | White | Not Hispanic or Latino | NA | 29 | 97 | 163 |
| **Average** | **37.0** | **-** | **-** | **-** | **-** | **26.6** | **92.9** | **180.4** |
| **XBB.1.5 MV (n=14)** | | | | | | | | |
| XBB.1.5-1 | 55 | Female | White | Not Hispanic or Latino | NA | 25 | 82 | 165 |
| XBB.1.5-2 | 38 | Male | White | Not Hispanic or Latino | NA | 27 | 88 | 171 |
| XBB.1.5-4 | 36 | Male | White | Not Hispanic or Latino | NA | 26 | 94 | 164 |
| XBB.1.5-5 | 58 | Female | Black | Not Hispanic or Latino | NA | 21 | 63 | 136 |
| XBB.1.5-6 | 60 | Female | White | Not Hispanic or Latino | NA | 25 | 82 | 223 |
| XBB.1.5-7 | 53 | Female | White | Not Hispanic or Latino | NA | 23 | 70 | 139 |
| XBB.1.5-8 | 27 | Female | White | Not Hispanic or Latino | NA | 32 | 76 | 153 |
| XBB.1.5-9 | 50 | Male | White | Not Hispanic or Latino | NA | 32 | 88 | 190 |
| XBB.1.5-10 | 60 | Female | White | Not Hispanic or Latino | NA | 28 | 80 | 178 |
| XBB.1.5-11 | 42 | Female | White | Not Hispanic or Latino | NA | 23 | 74 | 153 |
| XBB.1.5-12 | 58 | Female | White | Not Hispanic or Latino | NA | 21 | 71 | 155 |
| XBB.1.5-13 | 31 | Female | White | Not Hispanic or Latino | NA | 28 | 92 | 167 |
| XBB.1.5-14 | 68 | Male | White | Not Hispanic or Latino | NA | 27 | 85 | 176 |
| XBB.1.5-15 | 47 | Male | Asian | Not Hispanic or Latino | NA | 22 | 72 | 158 |
| **Average** | **48.8** | **-** | **-** | **-** | **-** | **25.7** | **79.8** | **166.3** |

**Table S3. Details of participants exposed to the BA.5 bivalent mRNA vaccine or the XBB.1.5 MV vaccine booster. Related to Figure 2.** Clinical information for each participant, including demographics and sample collection timepoints.

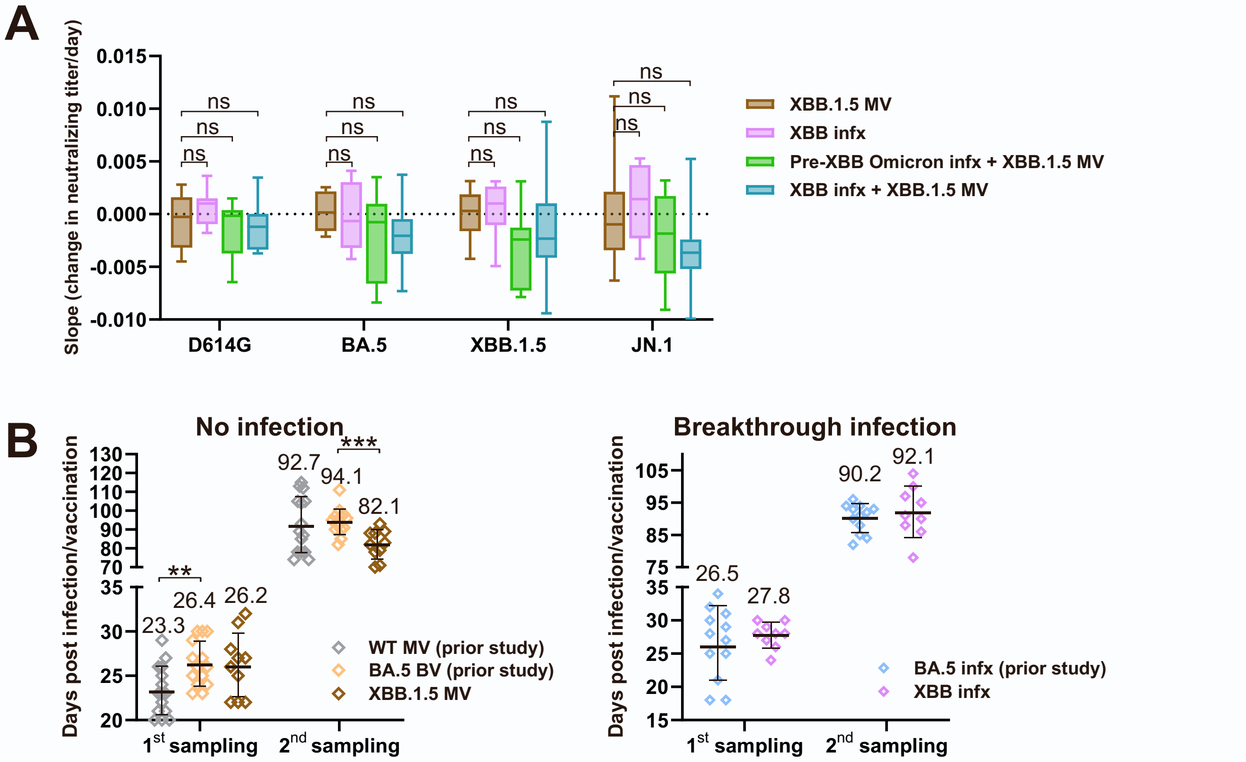


**Figure S1. $ID_{50}$ slope and sample collection days for the indicated cohorts, related to Figure 2.**

A. $ID_{50}$ slope (change in neutralizing titer per day) for the indicated cohorts.

B. Comparison of sample collection days post-vaccination or infection. Average days post-infection/vaccination marked above each group.

Statistical analyses were performed by Mann-Whitney U tests. ns, not significant; ***p*<0.01; ****p*<0.001.
