# Supplementary material for: Robust SARS-CoV-2-neutralizing antibodies sustained through 6 months post XBB.1.5 mRNA vaccine booster
Source: Cell Rep Med. 2024 Aug 28;5(9):101701. doi: 10.1016/j.xcrm.2024.101701 (PMC11524932; doi:10.1016/j.xcrm.2024.101701)
Supplement: Document S2. Article plus supplemental information [file mmc2.pdf]

Cell Reports
Medicine

Report

Robust SARS-CoV-2-neutralizing antibodies sustained through 6 months post XBB.1.5 mRNA vaccine booster

Graphical abstract

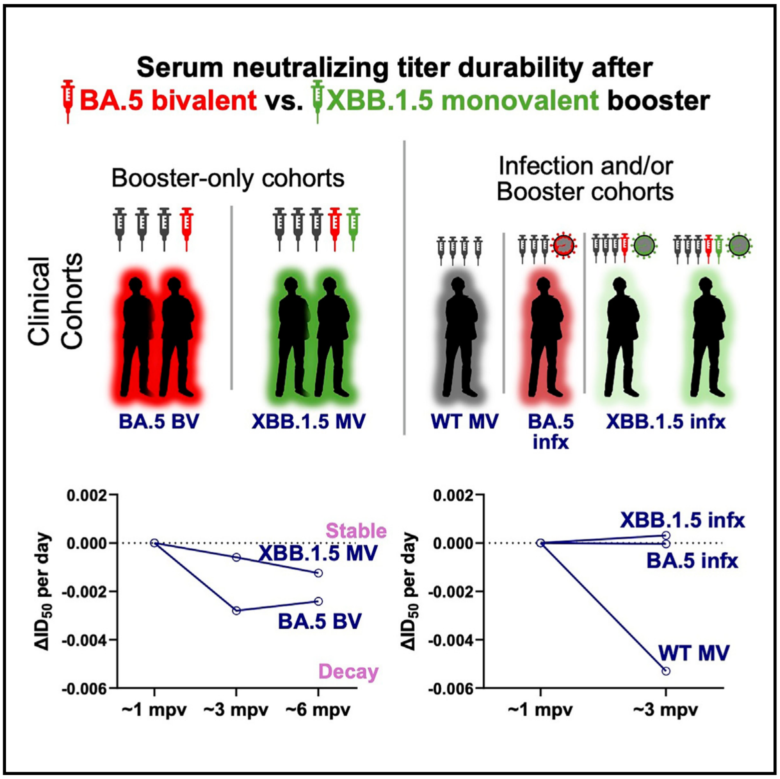

Highlights

- XBB.1.5 monovalent (MV) booster elicits neutralizing titers up to 6 months after dose
- Titers wane more slowly after XBB.1.5 MV booster than after BA.5 bivalent booster
- XBB.1.5 MV booster partially mitigates immunological imprinting

Authors

Qian Wang, Ian A. Mellis, Yicheng Guo, ..., Aubree Gordon, David D. Ho, Lihong Liu

Correspondence

gordonal@umich.edu (A.G.),
dh2994@cumc.columbia.edu (D.D.H.),
llh3411@whu.edu.cn (L.L.)

In brief

Wang and colleagues find that the XBB.1.5 vaccine booster yields robust serum SARS-CoV-2-neutralizing titers that are stable for ~6 months, in contrast to results after a booster with previous vaccines but consistent with recent national data showing protection against symptomatic infection lasts for 4 months after the updated vaccine booster.

Wang et al., 2024, Cell Reports Medicine *5*, 101701
September 17, 2024 © 2024 The Authors. Published by Elsevier Inc.
https://doi.org/10.1016/j.xcrm.2024.101701

CellPress

Cell Reports Medicine

CellPress
OPEN ACCESS

Report

Robust SARS-CoV-2-neutralizing antibodies sustained through 6 months post XBB.1.5 mRNA vaccine booster

Qian Wang,[1,2,10] Ian A. Mellis,[1,3,10] Yicheng Guo,[1,10] Carmen Gherasim,[4] Riccardo Valdez,[4] Aubree Gordon,[5,*] David D. Ho,[1,2,6,7,*] and Lihong Liu[8,9,11,*]

[1]Aaron Diamond AIDS Research Center, Columbia University Vagelos College of Physicians and Surgeons, New York, NY 10032, USA
[2]Pandemic Research Alliance Unit at the Wu Family Center, Columbia University Vagelos College of Physicians and Surgeons, New York, NY 10032, USA
[3]Department of Pathology and Cell Biology, Columbia University Vagelos College of Physicians and Surgeons, New York, NY 10032, USA
[4]Department of Pathology, University of Michigan, Ann Arbor, MI 48109, USA
[5]Department of Epidemiology, University of Michigan, Ann Arbor, MI 48109, USA
[6]Division of Infectious Diseases, Department of Medicine, Columbia University Vagelos College of Physicians and Surgeons, New York, NY 10032, USA
[7]Department of Microbiology and Immunology, Columbia University Vagelos College of Physicians and Surgeons, New York, NY 10032, USA
[8]State Key Laboratory of Virology, College of Life Sciences, Wuhan University, Wuhan, China
[9]Taikang Center for Life and Medical Sciences, Wuhan University, Wuhan, China
[10]These authors contributed equally
[11]Lead contact
*Correspondence: gordonal@umich.edu (A.G.), dh2994@cumc.columbia.edu (D.D.H.), llh3411@whu.edu.cn (L.L.)
https://doi.org/10.1016/j.xcrm.2024.101701

SUMMARY

Severe acute respiratory syndrome coronavirus 2 (SARS-CoV-2)-neutralizing antibodies are substantially expanded 1 month after a shot of XBB.1.5 monovalent mRNA vaccine (XBB.1.5 MV) booster, but the durability of this response remains unknown. Here, we address this question by performing neutralization assays on four viral variants (D614G, BA.5, XBB.1.5, and JN.1) using sera from participants obtained at ∼1 month, ∼3 months, and ∼6 months post an XBB.1.5 MV booster. Our findings indicate that the resulting neutralizing antibody titers are robust and generally remain at stable levels for the study period, similar to those following XBB infection. Importantly, this durability of neutralizing antibody titers contrasts with the decline observed after a booster of the original monovalent or BA.5 bivalent mRNA vaccine. Our results are in line with the recent national data from the Centers for Disease Control and Prevention, showing that the efficacy against symptomatic SARS-CoV-2 infection is sustained for up to 4 months after an XBB.1.5 MV booster.

INTRODUCTION

In the Fall of 2023, the United States Food and Drug Administration authorized new COVID-19 vaccines to replace the previous bivalent vaccines.[1] The updated vaccines target the spike protein of severe acute respiratory syndrome coronavirus 2 (SARS-CoV-2) Omicron subvariant XBB.1.5, aiming to enhance protection against the viral variant that was most dominant at the time strain selection was made.[2] Moreover, the updated vaccines were monovalent, because the prior ancestral/BA.5 bivalent vaccines did not appreciably expand the breadth of virus-neutralizing antibodies due to immunological imprinting that biased immune responses toward the ancestral strain.[3–7] We and others have since demonstrated that new updated XBB.1.5 mRNA monovalent vaccines (XBB.1.5 MV) boosted the potency and breadth of serum-neutralizing antibodies against not only XBB.1.5 but also other Omicron subvariants, including JN.1, approximately 1 month after administration.[2,8–15] The removal of the ancestral spike from the new vaccine formulations had seemingly mitigated but not eliminated the immunological imprinting observed with the previous bivalent vaccines. However, how the serum antibody responses evolve in the ensuing months after an XBB.1.5 MV booster remains unknown.

RESULTS

First, we addressed this question by evaluating the serum virus-neutralizing titers at two distinct time points in 39 participants distributed across four cohorts: (1) who had received an XBB.1.5 MV booster without history of SARS-CoV-2 infection (XBB.1.5 MV); (2) who had XBB sublineage virus infection without history of XBB.1.5 MV booster (XBB infection [XBB infx]); (3) who had received an XBB.1.5 MV booster with a prior pre-XBB Omicron infection history (pre-XBB Omicron infx + XBB.1.5 MV); and (4) who had received an XBB.1.5 MV booster with a prior XBB sublineage infection history (XBB infx + XBB.1.5 MV) (Figure 1A).

© 2024 The Authors. Published by Elsevier Inc.
This is an open access article under the CC BY license (http://creativecommons.org/licenses/by/4.0/).

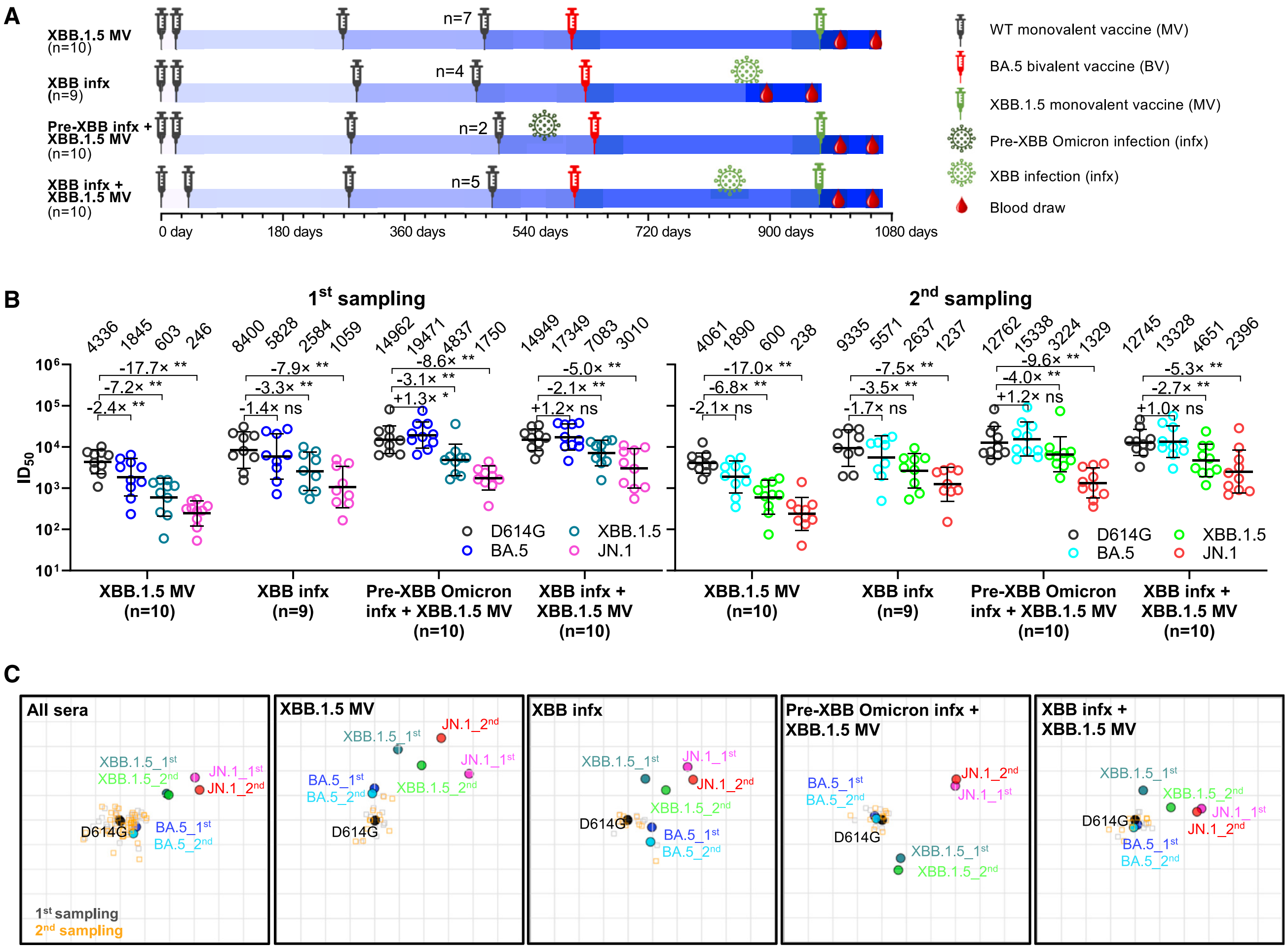

Figure 1. Neutralizing antibody titers in the months after an XBB.1.5 mRNA booster, XBB infection, or both

(A) Timelines of vaccine administration, SARS-CoV-2 infection, and serum collection intervals for the four clinical cohorts in this study. Indicated time points represent the mean in days since first SARS-CoV-2 vaccination for each participant; day 0 is defined as the day of the initial SARS-CoV-2 vaccination. All participants previously received 3–4 doses of wild-type (WT) monovalent vaccines (MV), followed by one dose of the BA.5 bivalent vaccine (BV) booster. Numbers of participants for each group receiving a fourth WT MV are indicated. Serum samples were collected from two time points after an XBB.1.5 MV booster or XBB sublineage infection, as indicated, and the mean sample collection days post vaccination or infection are summarized in Table S1. *n*, sample size.

(B) Serum virus-neutralizing titers (ID_{50}) of the four cohorts against the indicated SARS-CoV-2 pseudoviruses. Geometric mean ID_{50} titers (GMT) are shown along with the fold differences in GMT versus D614G. Statistical analyses comparing GMT between viruses were performed by Wilcoxon matched-pair signed rank tests. ns, not significant; $^{*}p < 0.05$; $^{**}p < 0.01$.

(C) Overlaid antigenic maps for serum virus-neutralizing titers at the first (∼26.4 days) and second (∼82.1 days) sampling time points. Panels in order: all cohorts, the XBB.1.5 monovalent vaccine (XBB.1.5 MV) cohort, the XBB infection (XBB infx) cohort, the pre-XBB infection + XBB.1.5 monovalent vaccine (pre-XBB infx + XBB.1.5 MV) cohort, and the XBB infection + XBB.1.5 monovalent vaccine (XBB infx + XBB.1.5 MV) cohort. Each panel contains two overlaid antigenic maps generated using sera from two time points post XBB exposure independently, with the D614G variant aligned. The x-y orientation of the component maps for either first sampling or second sampling is free, as only the relative distances between the variants within a sampling and the respective sampling's sera are compared. Distances between first sampling and second sampling variant points are not directly compared. One grid square on the antigenic maps corresponds to one antigenic unit, representing an approximately 2-fold change in ID_{50} titer. Variant positions are indicated by circles, while serum positions are denoted by gray squares (first sampling) or orange squares (second sampling). See also Tables S1 and S2.

The first and second serum samplings occurred at 26.4 and 82.1 mean days post vaccination or infection, respectively (Figure 1A; Table S1). Demographics and vaccination histories among the four clinical cohorts are presented in Tables S1 and S2. Serum-neutralizing antibody titers were determined using vesicular stomatitis virus-based pseudoviruses expressing the spike proteins of D614G (ancestral), BA.5, XBB.1.5, or JN.1.

Overall, similar patterns in neutralizing antibody titers (ID_{50}) were observed between serum samples from the first and second time points for all four cohorts (Figure 1B), with several features worthy of emphasis. First, neutralizing titers against all four viruses tested were robust (≥256) at both time points. Second, the highest neutralizing titers of the XBB.1.5 MV cohort were observed against D614G, while titers against XBB.1.5 were much lower (4,647 versus 646 and 4,535 versus 643 at first

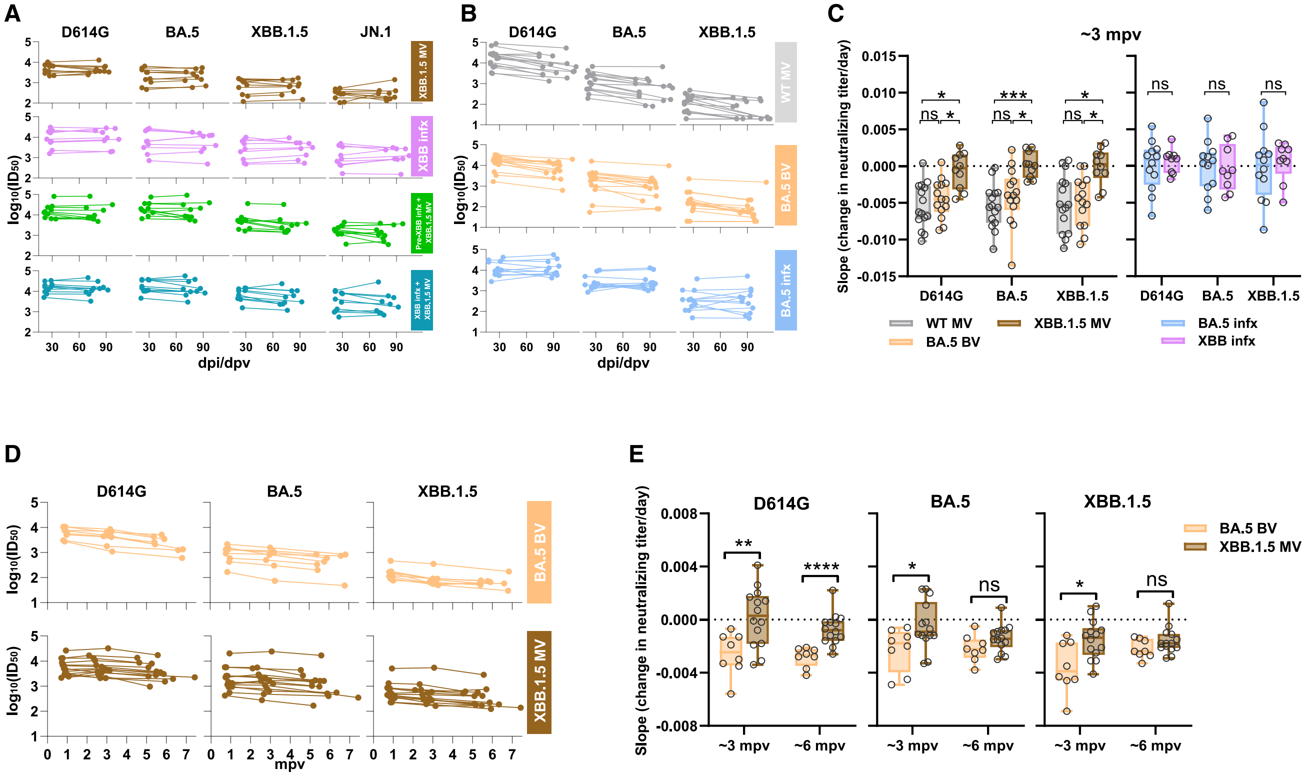

Figure 2. An XBB.1.5 monovalent vaccine booster induced sustained virus-neutralizing antibody responses through 6 months post vaccination

(A) Comparison of serum neutralizing antibody titers between two time points after XBB vaccination or infection for each participant in each cohort.

(B) Comparison of serum-neutralizing antibody titers between two time points after the fourth dose of the WT MV or BA.5 BV, or BA.5 breakthrough infection. Data for "WT MV," "BA.5 BV," and "BA.5 infx" cohorts were extracted from a previously published study.[4]

(C) Estimated change in neutralizing titer ($\log_{10}(ID_{50})$) per day (i.e., the slopes for serum samples collected from approximately 1 to 3 months) for cohorts without SARS-CoV-2 infection (left) or with breakthrough infection (right) across the two respective studies. Negative values indicate the rate of titer decay.

(D) Decline in neutralizing antibody titers over approximately a 6-month period in individuals who received either the XBB.1.5 monovalent vaccine booster or the BA.5 bivalent vaccine booster. mpv, month post vaccination.

(E) Estimated change in neutralizing titer ($\log_{10}(ID_{50})$) per day for longitudinal cohorts is presented in (D). Negative values indicate the rate of titer decay. Box-and-whisker plots, with whisker limits at minimum and maximum values and the central line representing median. Statistical analyses were performed by Mann-Whitney U tests. ns, not significant; *$p < 0.05$; **$p < 0.01$; ***$p < 0.001$; ****$p < 0.0001$. See also Figure S1 and Table S3.

and second time points, respectively). This finding showed the persistence of immunological back-boosting (also known as immunological imprinting) in eliciting neutralizing antibodies against SARS-CoV-2, although not as severe as previously observed for the ancestral/BA.5 bivalent vaccine booster.[3–7] Third, as expected, sera of participants from "pre-XBB Omicron infx + XBB.1.5 MV" and "XBB infx + XBB.1.5 MV" cohorts (individuals who had one more exposure to Omicron spike) showed substantially higher neutralizing titers against all four viruses. Fourth, sera from the XBB infx cohort exhibited stronger neutralizing activity (1.8- to 4.8-fold) than sera from the XBB.1.5 MV cohort. Lastly, among the viruses tested, serum-neutralizing titers were the lowest for JN.1 in all cohorts, indicating that it is the most antibody evasive, in line with our previous report.[2]

Antigenic maps were then generated using serum neutralization data from the four cohorts, both collectively and individually (Figure 1C), to visually summarize our findings. These maps showed that the antigenic distances between the ancestral D614G virus and other tested SARS-CoV-2 Omicron subvariants at both time points were consistent across all cohorts. Infections with an XBB sublineage virus seemed to slightly outperform a single XBB.1.5 MV booster in reducing antigenic distance. Additionally, having a history of Omicron infection prior to XBB.1.5 MV booster enhanced the neutralization of the currently dominant JN.1 subvariant. In particular, exposure to XBB sublineage infection before the booster appeared to reduce the antigenic distance to JN.1 by approximately one antigenic unit more than a pre-XBB Omicron infection.

We next endeavored to better understand the change in virus-neutralizing titers between the first and second time point serum samples. The ID_{50} titers against four viruses for all four cohorts were plotted using the actual dates of serum collection post vaccination or infection (Figure 2A). The daily decline in neutralizing titers (median slopes) is summarized in Figure S1A. No significant waning of neutralizing titers was observed for all four cohorts, except for a slight decrease in titer against XBB.1.5 in the pre-XBB infx + XBB.1.5 MV cohort (1.5-fold decrease). Additionally, no differences in antibody titer declines were found among all

four cohorts against each tested virus. However, in our prior study,[4] we observed around a 2-fold or greater decrease in neutralizing titers against D614G, BA.5, and XBB.1.5 from ~1 month to ~3 months post the fourth dose of the wild-type monovalent vaccine (WT MV) booster or ancestral/BA.5 bivalent vaccine (BV) booster, while a BA.5 breakthrough infection led to stable serum-neutralizing activity over the same interval (Figure 2B). Since the sample collection times across the two studies were slightly different (Table S1; Figure S1B), the most appropriate comparison of antibody decline would be to determine the slope between the $\log_{10}(ID_{50})$ titers for the two time points. Comparing cohorts with vaccine boosters but without any history or laboratory evidence of prior SARS-CoV-2 infection, the median slope for all four viruses tested was nearly zero (flat) after an XBB.1.5 MV booster in the present study, whereas the median slopes were significantly more negative (decline, with median values ranging from −0.0040/day to −0.0063/day) after a WT MV or ancestral/BA.5 BV booster in a prior study (Figure 2C). On the other hand, there was no appreciable decline during the study period for serum-neutralizing titers for individuals who had a BA.5 or XBB breakthrough infection in both studies (Figure 2C).

Lastly, in order to track antibody dynamics through time, we conducted a follow-up study, extending to approximately 6 months, for the exposure histories of two specific groups: 14 individuals vaccinated with an XBB.1.5 monovalent vaccine booster and 8 individuals with a BA.5 bivalent vaccine booster. Due to the limited availability of samples, most XBB.1.5 MV samples were obtained from additional individuals enrolled in a related study. Clinical details for all participants in the 6-month analysis are listed in Table S3. Consistent with the observation from the 3-month samples, the waning of titers until the 6-month time point was slower for those receiving the XBB.1.5 monovalent vaccine than for those receiving the BA.5 bivalent vaccine (Figures 2D and 2E).

DISCUSSION

In summary, an XBB.1.5 MV booster has elicited robust and sustained neutralizing antibodies against SARS-CoV-2, including the currently dominant JN.1 subvariant, for several months (Figures 1 and 2). The reduced waning of neutralizing antibodies is in distinct contrast to that observed after an ancestral MV or ancestral/BA.5 BV booster (Figure 2C). A clear explanation for this discrepancy is lacking. There is no indication that we may have missed the peak antibody response with the first serum sampling (Figure S1B). While an SARS-CoV-2 infection between the two time points could not be excluded, antibodies directed to the viral nucleocapsid could not be detected in the sera from the XBB.1.5 MV cohort (data not shown), nor is there a clinical history consistent with an intervening infection. The short-term durability of the potency and breadth of the virus-neutralization response following XBB.1.5 MV booster again suggests that immunological imprinting has been partially mitigated, likely due to the exclusion of the ancestral spike from the updated vaccine formulation. However, it should be noted that immunological imprinting has not been completely overridden by two exposures to an Omicron spike, as evident by higher neutralizing antibody titers against D614G compared to XBB.1.5 in the two cohorts that had an Omicron infection followed by an XBB.1.5 MV booster (Figure 1B). Importantly, our data are in agreement with vaccine effectiveness estimates recently reported by the Center for Disease Control and Prevention, showing no appreciable decline in protection against symptomatic COVID-19 up to 4 months post booster with updated vaccines.[16]

Limitations of the study

There are several notable limitations to this study. First, the number of study subjects in each cohort is relatively small, even though statistically significant results are reported. Second, we still lack an explanation for the mechanism by which neutralizing antibody titers after an XBB.1.5 MV booster are more stable than those elicited by earlier vaccinations. More studies are required to provide an answer. Third, the antibody responses assessed here, including back-boosting of titers against earlier SARS-CoV-2 variants, are exclusively serum-neutralizing antibodies. It would be beneficial for future research to encompass a variety of studies, such as antibody binding, memory B cell assays, and T cell analysis, to shed light on the specific mechanisms underlying immunological imprinting.

STAR★METHODS

Detailed methods are provided in the online version of this paper and include the following:

- KEY RESOURCES TABLE
- RESOURCE AVAILABILITY
  - Lead contact
  - Materials availability
  - Data and code availability
- EXPERIMENTAL MODEL AND SUBJECT DETAILS
  - Clinical cohorts
  - Cell lines
- METHOD DETAILS
  - Pseudovirus neutralization assay
  - Antigenic cartography
- QUANTIFICATION AND STATISTICAL ANALYSIS

SUPPLEMENTAL INFORMATION

Supplemental information can be found online at https://doi.org/10.1016/j.xcrm.2024.101701.

ACKNOWLEDGMENTS

This study was supported by funding from the NIH SARS-CoV-2 Assessment of Viral Evolution (SAVE) Program (subcontract no. 0258-A709-4609 under federal contract no. 75N93021C00014) and the Gates Foundation (project INV019355) to D.D.H., as well as funding from the NIH contracts 75N93019C00051 and 75N93021C00016 to A.G. and internal startup funding UR014016 from Columbia University to Y.G. We would also like to thank the Taikang Center for Life and Medical Sciences at Wuhan University, Wuhan, China, for providing funding support to L.L. We express our gratitude to Zijin Chu, Theresa Kowalski-Dobson, Emily Stoneman, David Manthei, Anna Buswinka, Gabe Simjanovski, Joseph Wendzinski, Mayurika Patel, Kathleen Lindsey, Dawson Davis, Victoria Blanc, Savanna Sneeringer, and Pamela Bennett-Baker of the IASO study team for conducting the IASO study.

AUTHOR CONTRIBUTIONS

L.L., A.G., and D.D.H. conceived and supervised this project. Q.W. managed the project. Q.W., I.A.M., and L.L. conducted pseudovirus neutralization assays. Y.G. and I.A.M. conducted bioinformatic analyses. C.G., R.V., and

A.G. provided clinical samples. Q.W., I.A.M., Y.G., L.L., and D.D.H. analyzed the results and wrote the manuscript. All authors reviewed the results and approved the final version of the manuscript.

DECLARATION OF INTERESTS

D.D.H. is a co-founder of TaiMed Biologics and RenBio, consultant to WuXi Biologics and Brii Biosciences, and board director for Vicarious Surgical. A.G. served on a scientific advisory board for Janssen Pharmaceuticals.

Received: February 26, 2024
Revised: May 20, 2024
Accepted: August 5, 2024
Published: August 28, 2024

REFERENCES

1. FDA (2023). Updated COVID-19 Vaccines for Use in the United States Beginning in Fall 2023. https://www.fda.gov/vaccines-blood-biologics/updated-covid-19-vaccines-use-united-states-beginning-fall-2023.
2. Wang, Q., Guo, Y., Bowen, A., Mellis, I.A., Valdez, R., Gherasim, C., Gordon, A., Liu, L., and Ho, D.D. (2024). XBB.1.5 monovalent mRNA vaccine booster elicits robust neutralizing antibodies against XBB subvariants and JN.1. Cell Host Microbe *32*, 315–321.e3. https://doi.org/10.1016/j.chom.2024.01.014.
3. Wang, Q., Bowen, A., Valdez, R., Gherasim, C., Gordon, A., Liu, L., and Ho, D.D. (2023). Antibody Response to Omicron BA.4-BA.5 Bivalent Booster. N. Engl. J. Med. *388*, 567–569. https://doi.org/10.1056/NEJMc2213907.
4. Wang, Q., Bowen, A., Tam, A.R., Valdez, R., Stoneman, E., Mellis, I.A., Gordon, A., Liu, L., and Ho, D.D. (2023). SARS-CoV-2 neutralising antibodies after bivalent versus monovalent booster. Lancet Infect. Dis. *23*, 527–528. https://doi.org/10.1016/S1473-3099(23)00181-0.
5. Wang, Q., Guo, Y., Tam, A.R., Valdez, R., Gordon, A., Liu, L., and Ho, D.D. (2023). Deep immunological imprinting due to the ancestral spike in the current bivalent COVID-19 vaccine. Cell Rep. Med. *4*, 101258. https://doi.org/10.1016/j.xcrm.2023.101258.
6. Wang, Q., Bowen, A., Ho, J., Zhang, R.M., Valdez, R., Stoneman, E., Gordon, A., Liu, L., and Ho, D.D. (2023). SARS-CoV-2 neutralising antibodies after a second BA.5 bivalent booster. Lancet *402*, 1827–1828. https://doi.org/10.1016/S0140-6736(23)02278-X.
7. Collier, A.R.Y., Miller, J., Hachmann, N.P., McMahan, K., Liu, J., Bondzie, E.A., Gallup, L., Rowe, M., Schonberg, E., Thai, S., et al. (2023). Immunogenicity of BA.5 Bivalent mRNA Vaccine Boosters. N. Engl. J. Med. *388*, 565–567. https://doi.org/10.1056/NEJMc2213948.
8. Kosugi, Y., Kaku, Y., Hinay, A.A., Jr., Guo, Z., Uriu, K., Kihara, M., Saito, F., Uwamino, Y., Kuramochi, J., Shirakawa, K., et al. (2024). Antiviral humoral immunity against SARS-CoV-2 omicron subvariants induced by XBB.1.5 monovalent vaccine in infection-naive and XBB-infected individuals. Lancet Infect. Dis. *24*, e147–e148. https://doi.org/10.1016/S1473-3099(23)00784-3.
9. Patel, N., Trost, J.F., Guebre-Xabier, M., Zhou, H., Norton, J., Jiang, D., Cai, Z., Zhu, M., Marchese, A.M., Greene, A.M., et al. (2023). XBB.1.5 spike protein COVID-19 vaccine induces broadly neutralizing and cellular immune responses against EG.5.1 and emerging XBB variants. Sci. Rep. *13*, 19176. https://doi.org/10.1038/s41598-023-46025-y.
10. Stankov, M.V., Hoffmann, M., Gutierrez Jauregui, R., Cossmann, A., Morillas Ramos, G., Graalmann, T., Winter, E.J., Friedrichsen, M., Ravens, I., Ilievska, T., et al. (2024). Humoral and cellular immune responses following BNT162b2 XBB.1.5 vaccination. Lancet Infect. Dis. *24*, e1–e3. https://doi.org/10.1016/S1473-3099(23)00690-4.
11. Jain, S., Kumar, S., Lai, L., Linderman, S., Malik, A.A., Ellis, M.L., Godbole, S., Solis, D., Sahoo, M.K., Bechnak, K., et al. (2024). XBB.1.5 monovalent booster improves antibody binding and neutralization against emerging SARS-CoV-2 Omicron variants. Preprint at bioRxiv. https://doi.org/10.1101/2024.02.03.578771.
12. Liang, C.-Y., Raju, S., Liu, Z., Li, Y., Arunkumar, G.A., Case, J.B., Zost, S.J., Acreman, C.M., Carvalho dos Anjos, D.C., McLellan, J.S., et al. (2024). Prototype mRNA vaccines imprint broadly neutralizing human serum antibodies after Omicron variant-matched boosting. Preprint at bioRxiv. https://doi.org/10.1101/2024.01.03.574018.
13. Chalkias, S., McGhee, N., Whatley, J.L., Essink, B., Brosz, A., Tomassini, J.E., Girard, B., Edwards, D.K., Wu, K., Nasir, A., et al. (2024). Interim report of the reactogenicity and immunogenicity of SARS-CoV-2 XBB-containing vaccines. J. Infect. Dis., jiae067. https://doi.org/10.1093/infdis/jiae067.
14. Modjarrad, K., Che, Y., Chen, W., Wu, H., Cadima, C.I., Muik, A., Maddur, M.S., Tompkins, K.R., Martinez, L.T., Cai, H., et al. (2023). Preclinical Characterization of the Omicron XBB.1.5-Adapted BNT162b2 COVID-19 Vaccine. Preprint at bioRxiv. https://doi.org/10.1101/2023.11.17.567633.
15. Marking, U., Bladh, O., Aguilera, K., Yang, Y., Greilert Norin, N., Blom, K., Hober, S., Klingström, J., Havervall, S., Åberg, M., et al. (2024). Humoral immune responses to the monovalent XBB.1.5-adapted BNT162b2 mRNA booster in Sweden. Lancet Infect. Dis. *24*, e80–e81. https://doi.org/10.1016/S1473-3099(23)00779-X.
16. Link-Gelles, R., Ciesla, A.A., Mak, J., Miller, J.D., Silk, B.J., Lambrou, A.S., Paden, C.R., Shirk, P., Britton, A., Smith, Z.R., and Fleming-Dutra, K.E. (2024). Early Estimates of Updated 2023-2024 (Monovalent XBB.1.5) COVID-19 Vaccine Effectiveness Against Symptomatic SARS-CoV-2 Infection Attributable to Co-Circulating Omicron Variants Among Immunocompetent Adults - Increasing Community Access to Testing Program, United States, September 2023-January 2024. MMWR Morb. Mortal. Wkly. Rep. *73*, 77–83. https://doi.org/10.15585/mmwr.mm7304a2.
17. Wang, Q., Guo, Y., Iketani, S., Nair, M.S., Li, Z., Mohri, H., Wang, M., Yu, J., Bowen, A.D., Chang, J.Y., et al. (2022). Antibody evasion by SARS-CoV-2 Omicron subvariants BA.2.12.1, BA.4 and BA.5. Nature *608*, 603–608. https://doi.org/10.1038/s41586-022-05053-w.
18. Simon, V., Kota, V., Bloomquist, R.F., Hanley, H.B., Forgacs, D., Pahwa, S., Pallikkuth, S., Miller, L.G., Schaenman, J., Yeaman, M.R., et al. (2022). PARIS and SPARTA: Finding the Achilles' Heel of SARS-CoV-2. mSphere *7*, e0017922. https://doi.org/10.1128/msphere.00179-22.
19. Smith, D.J., Lapedes, A.S., de Jong, J.C., Bestebroer, T.M., Rimmelzwaan, G.F., Osterhaus, A.D.M.E., and Fouchier, R.A.M. (2004). Mapping the antigenic and genetic evolution of influenza virus. Science *305*, 371–376. https://doi.org/10.1126/science.1097211.

STAR★METHODS

KEY RESOURCES TABLE

REAGENT or RESOURCE	SOURCE	IDENTIFIER
Bacterial and virus strains		
VSV-G pseudotyped ΔG-luciferase	Kerafast	Cat# EH1020-PM
Biological samples		
"XBB.1.5 MV" sera	This paper	N/A
"XBB infx" sera	This paper	N/A
"Pre-XBB infx + XBB.1.5 MV" sera	This paper	N/A
"XBB infx + XBB.1.5 MV" sera	This paper	N/A
"WT MV" sera	Wang et al.[4]	N/A
"BA.5 MV" sera	This paper and Wang et al.[4]	N/A
Chemicals, peptides, and recombinant proteins		
Polyethylenimine (PEI)	Polysciences Inc.	Cat# 23966-100
Critical commercial assays		
Luciferase Assay System	Promega	Cat# E4550
Experimental models: Cell lines		
HEK293T	ATCC	Cat# CRL-3216; RRID: CVCL_0063
Vero-E6	ATCC	Cat# CRL-1586; RRID: CVCL_0574
Recombinant DNA		
pCMV3-D614G	Wang et al.[17]	N/A
pCMV3-BA.5	Wang et al.[17]	N/A
pCMV3-XBB.1.5	Wang et al.[5]	N/A
pCMV3-JN.1	Wang et al.[2]	N/A
Software and algorithms		
GraphPad Prism V.10	GraphPad Software Inc	https://www.graphpad.com/scientific-software/prism/

RESOURCE AVAILABILITY

Lead contact

Further information and requests for resources and reagents should be directed to and will be fulfilled by the lead contact, Lihong Liu (llh3411@whu.edu.cn).

Materials availability

All reagents generated in this study are available from the lead contact author with a completed Materials Transfer Agreement.

Data and code availability

Data:

Data reported in this paper will be shared by the lead contact author upon request.

Code:

This paper does not report original code. Any additional information required to reanalyze the data reported in this paper and R scripts (version 1.1.4, available at https://acorg.github.io/Racmacs/) used to perform most statistical analyses are available from the lead contact author upon request.

EXPERIMENTAL MODEL AND SUBJECT DETAILS

Clinical cohorts

Longitudinal sera were obtained as part of an ongoing cohort study, Immunity-Associated with SARS-CoV-2 Study (IASO), which began in 2020 at the University of Michigan in Ann Arbor, Michigan.[18] All participants provided written informed consent, and

sera were collected according to the protocol approved by the Institutional Review Board of the University of Michigan Medical School. Participants in the IASO study completed weekly symptom surveys, and if any symptoms were reported, participants were tested for SARS-CoV-2. We tested all serum samples by anti-nucleoprotein (NP) ELISA to check for any potential breakthrough infections during the period spanning sample collections for this study.

In this study, we included sera from 39 individuals across four clinical cohorts: 1) individuals with no recorded SARS-CoV-2 infections who had received an XBB.1.5 monovalent vaccine booster (“XBB.1.5 MV”); 2) individuals with a recent XBB sublineage infection who had not received the XBB.1.5 booster (“XBB infx); 3) individuals with pre-XBB Omicron infection who also received the XBB.1.5 booster (“Pre-XBB Omicron infx + XBB MV”); and 4) individuals with an XBB sublineage infection who also received the XBB.1.5 booster (XBB infx + XBB MV). Individuals in all cohorts previously received either three or four doses of a wildtype monovalent vaccine as well as a single ancestral/BA.5 bivalent booster. Most participants were female (79.5%) with an average age of 51.6 years. Sera were collected an average of 26.4 and 82.1 days after XBB.1.5 vaccination or XBB sublineage infection. Demographic, vaccination, and serum collection details are summarized for each cohort in Table S1, and details are shown for each participant in Table S2. Additionally, we collected longitudinal serum samples at approximately 1-month, 3-month, and 6-month intervals from 14 individuals vaccinated with an XBB.1.5 monovalent vaccine booster and 8 individuals with a BA.5 bivalent vaccine booster. Clinical details for all cases are provided in Table S3.

Cell lines

We obtained 293T (CRL-3216) and Vero-E6 (CRL-1586) cells from ATCC and cultured them in a humidified incubator at 37°C, supplemented with 5% CO_2. The morphology of each cell line was visually confirmed before use. All cell lines tested negative for mycoplasma. The research resource identifiers (RRIDs) for the cell lines used in our study are listed in the key resources table.

METHOD DETAILS

Pseudovirus neutralization assay

Plasmids encoding SARS-CoV-2 variant spikes, including D614G, BA.5, XBB.1.5, and JN.1, were generated in previous studies.[2,5,17]

To produce pseudotyped viruses of SARS-CoV-2 variants, we transfected 293T cells with the spike-encoding plasmids described above using 1 mg/mL PEI (Polyethylenimine). One day post-transfection, the 293T cells were incubated with VSVG*ΔG-luciferase (Kerafast, Inc.) at a multiplicity of infection of approximately 3–5 for 2 h followed by three washes with complete culture medium. The cells were then cultured with fresh medium for an additional day. Cell supernatants containing viruses were collected, clarified by centrifugation, aliquoted, and stored at −80°C until use.

The viral titer of each variant was titrated to calculate a 50% tissue-culture-infectious dose ($TCID_{50}$) and normalized for neutralization assays. Serum samples were diluted in triplicate in 96-well plates, starting from a 12.5-fold dilution (for one sample, a 50-fold starting dilution was necessary due to volume constraints), and then incubated with an equal volume of virus for 1 h at 37°C before adding 2×10^4 cells/well of Vero-E6 cells. The cells were then cultured overnight, harvested, and lysed for measurement of luciferase activity using SoftMax Pro v.7.0.2 (Molecular Devices). Reductions in luciferase activity at given dilutions of sera were calculated, and ID_{50} values of sera were obtained by fitting the virus-reduction data using a non-linear five-parameter dose-response curve in GraphPad Prism V.10.

Antigenic cartography

Antigenic maps for D614G and other SARS-CoV-2 variants were generated by integrating the ID_{50} values of individual serum samples using a published antigenic cartography method.[19] Visualizations were created with the Racmacs package (version 1.1.4, available at https://acorg.github.io/Racmacs/) in R software version 4.0.3. The optimization was performed over 2,000 steps, with the “minimum column basis” parameter set to “none”. The “mapDistances” function calculated the antigenic distances between each serum sample and each variant. D614G was used as center for each group’s sera, and the seeds for each antigenic map were manually adjusted to ensure that XBB.1.5 and JN.1 were oriented correctly relative to D614G. All maps are aligned with D614G positioned in the middle left, ensuring a consistent reference point across the maps.

QUANTIFICATION AND STATISTICAL ANALYSIS

Serum neutralization ID_{50} values were calculated using a five-parameter dose-response curve in GraphPad Prism v.10. Evaluations of statistical significance were performed employing either Wilcoxon matched-pairs signed rank tests or Mann-Whitney U tests using GraphPad Prism v.10 software.

Cell Reports Medicine, Volume *5*

Supplemental information

Robust SARS-CoV-2-neutralizing antibodies sustained through 6 months post XBB.1.5 mRNA vaccine booster

Qian Wang, Ian A. Mellis, Yicheng Guo, Carmen Gherasim, Riccardo Valdez, Aubree Gordon, David D. Ho, and Lihong Liu

Clinical Information		Across all		XBB.1.5 MV		XBB infx		Prior Omicron infection + XBB.1.5 MV			
								Pre-XBB infx + XBB.1.5 MV		XBB infx + XBB.1.5 MV	
		Number or mean	% or range	Number or mean	% or range	Number or mean	% or range	Number or mean	% or range	Number or mean	% or range
**Total participants**		39		10		9		10		10	
**Male**		8	20.5%	3	30%	1	11.10%	2	20%	2	20%
**Female**		31	79.5%	7	70%	8	88.90%	8	80%	8	80%
**Age**		51.6	(30, 67)	52.6	(38, 65)	52.1	(40, 59)	49.7	(36, 67)	52.1	(30, 62)
**First two WT doses**	**Pfizer**	35	89.7%	9	90%	8	88.90%	9	90%	9	90%
	**Moderna**	3	7.7%	1	10%	1	11.10%	1	10%	0	0%
	**Janssen**	1	2.6%	0	0%	0	0%	0	0%	1	10%
**Third WT dose**	**Pfizer**	34	87.2%	9	90%	8	88.90%	9	90%	8	80%
	**Moderna**	5	12.8%	1	10%	1	11.10%	1	10%	2	20%
**Fourth WT dose**	**Pfizer**	13	33.3%	4	40%	3	33.30%	2	20%	4	40%
	**Moderna**	5	12.8%	3	30%	1	11.10%	0	0%	1	10%
	**None**	21	53.8%	3	30%	5	55.60%	8	80%	5	50%
**BA.5 bivalent booster**	**Pfizer**	23	59%	6	60%	7	77.80%	4	40%	6	60%
	**Moderna**	16	41%	4	40%	2	22.20%	6	60%	4	40%
**XBB MV booster**	**Pfizer**	13	33.3%	4	40%	0	0%	3	30%	6	60%
	**Moderna**	17	43.6%	6	60%	0	0%	7	70%	4	40%
	**None**	9	23.1%	0	0%	9	100%	0	0%	0	0%
**First sample post-XBB (days)**		26.4	(21, 34)	26.2	(22, 32)	27.8	(24, 30)	26.9	(21, 34)	24.7	(21, 30)
**Second sample post-XBB (days)**		82.1	(68, 104)	82.1	(70, 93)	92.1	(78, 104)	77.6	(68, 97)	77.5	(68, 92)

Table S1. Summary of information on participants in this study who were exposed to XBB. Related to Figure 1. Demographic, vaccine, and serum collection information summarized for each cohort. Listed values represent the mean and range (age and sera collection) or number and percentage (vaccine type and sex).

Participant ID	Age	Gender	Race	Ethnicity	Infection Period	WT vaccines				Bivalent vaccine	XBB.1.5 MV vaccine	Days post-XBB		Days after 1st WT vaccine dose				
						1st dose	2nd dose	3rd dose	4th dose			1st sample	2nd sample	2nd WT dose	3rd WT dose	4th WT dose	Bivalent vaccine	XBB.1.5 MV vaccine
**XBB.1.5 MV (n=12)**																		
G4-1	62	Female	White	Not Hispanic or Latino	NA	Pfizer	Pfizer	Pfizer	Pfizer	Pfizer	Moderna	27	93	22	268	491	657	993
G4-4	65	Female	White	Not Hispanic or Latino	NA	Pfizer	Pfizer	Pfizer	Pfizer	Pfizer	Pfizer	28	79	22	266	503	637	990
G4-6	55	Female	White	Not Hispanic or Latino	NA	Pfizer	Pfizer	Pfizer	Moderna	Moderna	Moderna	25	82	21	272	472	637	995
G4-8	38	Male	White	Not Hispanic or Latino	NA	Moderna	Moderna	Moderna	NA	Moderna	Pfizer	27	88	28	235	NA	539	914
G4-9	64	Female	White	Not Hispanic or Latino	NA	Pfizer	Pfizer	Pfizer	Pfizer	Pfizer	Pfizer	32	70	21	270	455	606	976
G4-12	40	Male	White	Not Hispanic or Latino	NA	Pfizer	Pfizer	Pfizer	NA	Moderna	Moderna	22	78	21	268	NA	584	969
G4-14	50	Male	White	Not Hispanic or Latino	NA	Pfizer	Pfizer	Pfizer	Moderna	Pfizer	Moderna	31	88	21	235	444	621	996
G4-15	54	Female	White	Not Hispanic or Latino	NA	Pfizer	Pfizer	Pfizer	Moderna	Pfizer	Moderna	22	89	21	329	490	623	990
G4-17	56	Female	White	Not Hispanic or Latino	NA	Pfizer	Pfizer	Pfizer	Pfizer	Pfizer	Moderna	22	71	21	287	507	654	1016
G4-19	42	Female	White	Not Hispanic or Latino	NA	Pfizer	Pfizer	Pfizer	NA	Moderna	Pfizer	26	83	21	239	NA	520	910
**XBB infx (n=9)**																		
G3-4	59	Female	White	Not Hispanic or Latino	2023.08	Pfizer	Pfizer	Pfizer	Pfizer	Pfizer	NA	26	78	21	295	472	660	NA
G3-6	54	Female	White	Not Hispanic or Latino	2023.09	Pfizer	Pfizer	Pfizer	Pfizer	Pfizer	NA	28	100	21	222	421	602	NA
G3-9	59	Female	White	Not Hispanic or Latino	2023.05	Pfizer	Pfizer	Pfizer	Pfizer	Pfizer	NA	30	86	25	281	469	613	NA
G3-12	42	Male	White	Not Hispanic or Latino	2023.04	Pfizer	Pfizer	Pfizer	NA	Moderna	NA	28	88	21	269	NA	615	NA
G3-15	53	Female	White	Not Hispanic or Latino	2023.02	Pfizer	Pfizer	Pfizer	NA	Pfizer	NA	27	97	22	320	NA	645	NA
G3-16	40	Female	White	Not Hispanic or Latino	2023.05	Pfizer	Pfizer	Pfizer	NA	Pfizer	NA	28	90	21	279	NA	658	NA
G3-17	49	Female	White	Not Hispanic or Latino	2023.07	Pfizer	Pfizer	Pfizer	NA	Moderna	NA	30	95	22	328	NA	641	NA
G3-18	59	Female	White	Not Hispanic or Latino	2023.04	Moderna	Moderna	Moderna	Moderna	Pfizer	NA	24	91	28	268	505	584	NA
G3-20	54	Female	White	Not Hispanic or Latino	2023.08	Pfizer	Pfizer	Pfizer	NA	Pfizer	NA	29	104	23	284	NA	632	NA
**Pre-XBB infx + XBB.1.5 MV (n=10)**																		
G5-1	41	Female	Declined	Not Hispanic or Latino	2022.09	Moderna	Moderna	Moderna	NA	Pfizer	Moderna	25	68	28	330	NA	715	1009
G5-2	61	Female	White	Not Hispanic or Latino	2022.04	Pfizer	Pfizer	Pfizer	NA	Moderna	Moderna	34	76	22	302	NA	649	1006
G5-3	53	Female	White	Not Hispanic or Latino	2022.04	Pfizer	Pfizer	Pfizer	NA	Pfizer	Moderna	25	81	21	275	NA	626	999
G5-4	49	Male	White	Not Hispanic or Latino	2022.01	Pfizer	Pfizer	Pfizer	NA	Pfizer	Pfizer	22	77	21	265	NA	691	956
G5-5	67	Female	White	Not Hispanic or Latino	2022.07	Pfizer	Pfizer	Pfizer	Pfizer	Moderna	Moderna	29	73	21	271	473	690	992
G5-7	48	Female	White	Not Hispanic or Latino	2022.01	Pfizer	Pfizer	Pfizer	NA	Moderna	Pfizer	22	75	21	254	NA	554	925
G5-10	40	Female	Multiple	Not Hispanic or Latino	2022.04	Pfizer	Pfizer	Pfizer	NA	Moderna	Moderna	21	70	24	276	NA	638	1003
G5-12	43	Female	White	Not Hispanic or Latino	2022.01	Pfizer	Pfizer	Pfizer	NA	Moderna	Pfizer	32	72	21	234	NA	566	913
G5-13	36	Male	White	Not Hispanic or Latino	2022.08	Pfizer	Pfizer	Pfizer	NA	Moderna	Moderna	30	97	21	316	NA	655	980
G5-14	59	Female	White	Not Hispanic or Latino	2022.04	Pfizer	Pfizer	Pfizer	Pfizer	Pfizer	Moderna	29	87	21	277	543	610	986
**XBB infx + XBB.1.5 MV (n=10)**																		
G6-1	54	Female	White	Not Hispanic or Latino	2023.02	Pfizer	Pfizer	Pfizer	Moderna	Pfizer	Moderna	22	70	21	262	492	632	1003
G6-2	58	Female	White	Not Hispanic or Latino	2023.02	Pfizer	Pfizer	Pfizer	NA	Pfizer	Pfizer	22	74	21	284	NA	682	1000
G6-3	61	Male	White	Not Hispanic or Latino	2023.05	Pfizer	Pfizer	Moderna	NA	Moderna	Moderna	30	87	21	231	NA	541	917
G6-4	42	Female	White	Not Hispanic or Latino	2023.03	Pfizer	Pfizer	Pfizer	Pfizer	Moderna	Pfizer	29	83	21	266	477	608	996
G6-5	62	Female	White	Not Hispanic or Latino	2023.06	Pfizer	Pfizer	Pfizer	Pfizer	Pfizer	Pfizer	21	73	21	277	439	643	1002
G6-6	46	Male	White	Not Hispanic or Latino	2023.05	Janssen	Janssen	Moderna	NA	Pfizer	Pfizer	24	71	214	386	NA	543	921
G6-7	61	Female	White	Not Hispanic or Latino	2023.04	Pfizer	Pfizer	Pfizer	Pfizer	Moderna	Moderna	26	76	21	275	519	625	1004
G6-8	62	Female	Asian	Not Hispanic or Latino	2023.04	Pfizer	Pfizer	Pfizer	Pfizer	Pfizer	Moderna	29	81	21	294	516	630	1000
G6-9	30	Female	White	Hispanic or Latino	2023.08	Pfizer	Pfizer	Pfizer	NA	Moderna	Pfizer	22	68	21	218	NA	580	922
G6-11	45	Female	White	Not Hispanic or Latino	2023.08	Pfizer	Pfizer	Pfizer	NA	Pfizer	Pfizer	22	92	21	269	NA	624	984

Table S2. Details of participants exposed to XBB. Related to Figure 1. Clinical information for each participant, including demographic, vaccine, infection, and sera details.

Participant ID	Age	Gender	Race	Ethnicity	Infection Period	Days post-vaccination		
						1st sample	2nd sample	3rd sample
**BA.5 BV (n=8)**								
BA.5-1	24	Female	White	Not Hispanic or Latino	NA	30	85	162
BA.5-2	43	Female	White	Not Hispanic or Latino	NA	26	91	205
BA.5-3	45	Male	White	Not Hispanic or Latino	NA	26	98	204
BA.5-4	36	Female	White	Not Hispanic or Latino	NA	27	90	173
BA.5-5	38	Female	White	Not Hispanic or Latino	NA	24	90	198
BA.5-6	36	Male	White	Not Hispanic or Latino	NA	24	95	161
BA.5-7	42	Female	White	Not Hispanic or Latino	NA	27	97	177
BA.5-8	32	Female	White	Not Hispanic or Latino	NA	29	97	163
**Average**	**37.0**	**-**	**-**	**-**	**-**	**26.6**	**92.9**	**180.4**
**XBB.1.5 MV (n=14)**								
XBB.1.5-1	55	Female	White	Not Hispanic or Latino	NA	25	82	165
XBB.1.5-2	38	Male	White	Not Hispanic or Latino	NA	27	88	171
XBB.1.5-4	36	Male	White	Not Hispanic or Latino	NA	26	94	164
XBB.1.5-5	58	Female	Black	Not Hispanic or Latino	NA	21	63	136
XBB.1.5-6	60	Female	White	Not Hispanic or Latino	NA	25	82	223
XBB.1.5-7	53	Female	White	Not Hispanic or Latino	NA	23	70	139
XBB.1.5-8	27	Female	White	Not Hispanic or Latino	NA	32	76	153
XBB.1.5-9	50	Male	White	Not Hispanic or Latino	NA	32	88	190
XBB.1.5-10	60	Female	White	Not Hispanic or Latino	NA	28	80	178
XBB.1.5-11	42	Female	White	Not Hispanic or Latino	NA	23	74	153
XBB.1.5-12	58	Female	White	Not Hispanic or Latino	NA	21	71	155
XBB.1.5-13	31	Female	White	Not Hispanic or Latino	NA	28	92	167
XBB.1.5-14	68	Male	White	Not Hispanic or Latino	NA	27	85	176
XBB.1.5-15	47	Male	Asian	Not Hispanic or Latino	NA	22	72	158
**Average**	**48.8**	**-**	**-**	**-**	**-**	**25.7**	**79.8**	**166.3**

Table S3. Details of participants exposed to the BA.5 bivalent mRNA vaccine or the XBB.1.5 MV vaccine booster. Related to Figure 2. Clinical information for each participant, including demographics and sample collection timepoints.

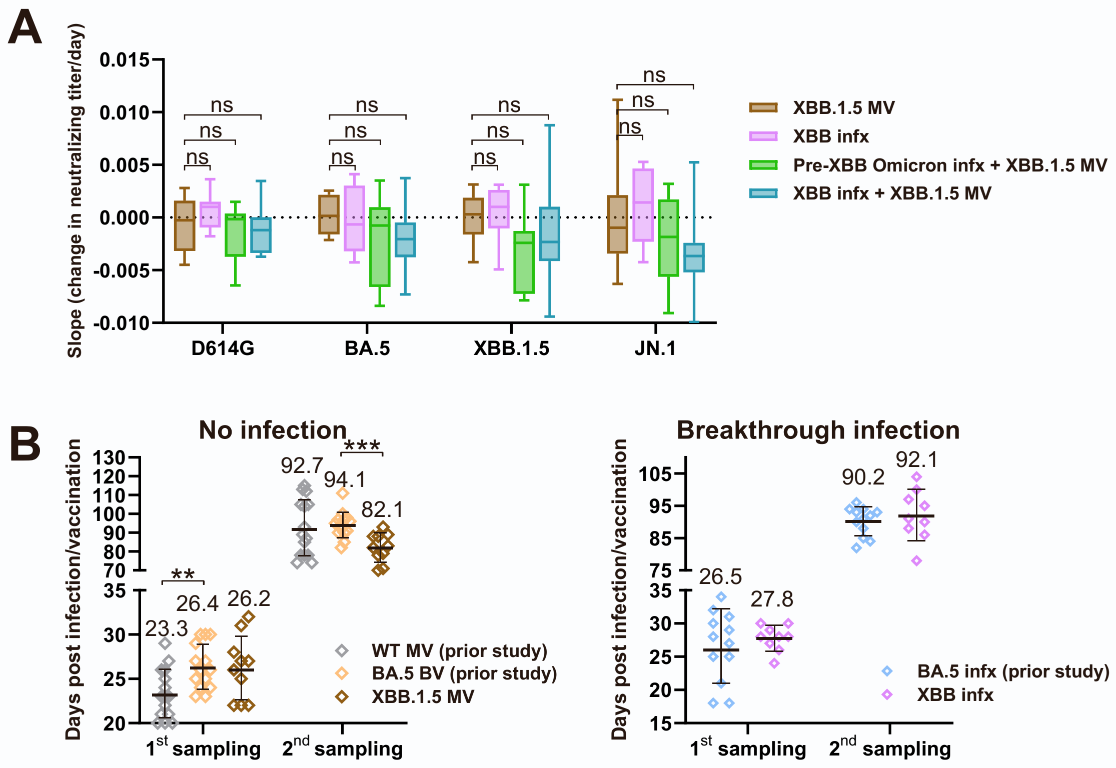

Figure S1. ID_{50} slope and sample collection days for the indicated cohorts, related to Figure 2.

A. ID_{50} slope (change in neutralizing titer per day) for the indicated cohorts.

B. Comparison of sample collection days post-vaccination or infection. Average days post-infection/vaccination marked above each group.

Statistical analyses were performed by Mann-Whitney U tests. ns, not significant; $^{**}p<0.01$; $^{***}p<0.001$.
